# Sustainable biosynthesis of silver nanoparticles from vinegar bacteria fermentation waste: characterization, bioactivity and food packaging potential

**DOI:** 10.1038/s41598-026-53384-9

**Published:** 2026-05-14

**Authors:** Perihan Akbaş, Ali Osman Adıgüzel, Elife Kaya, Serpil Könen Adıgüzel, Eda Uğurtay

**Affiliations:** 1 Karadeniz Advanced Technology Research and Application Center, Ondokuz Mayıs University, Samsun, Turkey; 2 Department of Molecular Biology and Genetics, Faculty of Science, Ondokuz Mayıs University, Samsun, Turkey; 3 Stem Cell Research and Application Center, Ondokuz Mayıs University, Samsun, Turkey; 4 Department of Food Processing, Technical Sciences Vocational School, Kahramanmaraş Sütçü Imam University, Kahramanmaraş, Turkey

**Keywords:** Acetic acid bacteria, Antimicrobial, Antioxidant, Cytotoxicity, Green synthesis, Silver nanoparticles, Biochemistry, Biological techniques, Biotechnology, Microbiology, Nanoscience and technology

## Abstract

Silver nanoparticles (AgNPs) have emerged as promising agents with significant potential for biotechnological applications, particularly in active packaging systems and wound-healing dressings, owing to their broad-spectrum antimicrobial activity and ability to enhance product safety, shelf life, and infection control. In this context, AgNPs were successfully synthesized via a green synthesis method, offering an environmentally friendly, sustainable, and low-cost approach, using *Acetobacter* sp. (strains 1A, 1B, 2B, 2D, 3A) and *Komagataeibacter* sp. (strain X1) isolated from homemade vinegars in the province of Samsun. The synthesis of nanoparticles was verified by the presence of surface plasmon resonance (SPR) peaks formed at 414–432 nm in the reaction mixture. The scanning electron microscopy (SEM) analysis showed that AgNPs were spherical in shape and varied in size between 20 and 100 nm depending on the bacterial isolate used in the synthesis. DLS analysis revealed that all synthesized AgNPs exhibited hydrodynamic diameters ranging from 85.05 to 179.1 nm with PDI values between 0.274 and 0.402, indicating moderately monodisperse to heterogeneous distributions depending on the bacterial isolate. Zeta potential measurements demonstrated that AgNPs had negatively charged surfaces with moderate to high colloidal electrostatic stability (− 12.3 to − 31.8 mV). The antimicrobial activity of the obtained AgNPs was tested against Gram-negative and Gram-positive bacteria, with a low inhibition concentration (16 µg/mL) particularly observed in the ESBL-positive *E. coli* strain. Antioxidant tests revealed that AgNPs synthesized using *Komagataeibacter* sp. X1 (KX1-AgNP) exhibited the highest activity and effectively neutralized DPPH radicals by 56.01%. Cytotoxicity studies showed that AgNPs preserved fibroblast cell viability at low concentrations but exhibited toxic effects at high doses. Packaging analyses showed that X1-film effectively preserved food by significantly reducing total viable, psychrotrophic, and yeast–mold counts during storage. These results indicate that biosynthesized AgNPs possess strong antimicrobial and antioxidant properties, with vinegar bacteria serving as an efficient, eco-friendly, and cost-effective source. This strategy offers promising potential for sustainable applications in food packaging and nanotechnology.

**Supplementary Information:**

The online version contains supplementary material available at 10.1038/s41598-026-53384-9.

## Introduction

In recent years, nanotechnology has led to revolutionary developments in many fields, particularly medicine, and the environment and agricultural nanoscale materials have at least one dimension between 1 and 100 nm. Owing to their small size, they provide special benefits in terms of functional qualities, interactions with biological systems, and high surface-to-volume ratios^1^. Silver nanoparticles are metallic nanoparticles widely used in nanotechnology due to their antimicrobial, anti-inflammatory, anticancer, and antioxidant properties^2,3^. Because of these characteristics, AgNPs are appealing for a variety of uses, including wound healing materials, biosensors, drug delivery systems, and food packaging^4^.

Physical and chemical methods for producing metal nanoparticles often involve toxic chemicals, are energy intensive, and generate environmentally harmful waste^5^. Biological methods, also referred to as ‘green synthesis,’ are increasingly recognized for their utilization of environmentally friendly reagents and the generation of reduced toxic waste^6,7^. Microorganisms are considered effective biocatalysts in nanoparticle synthesis because of their ability to biologically reduce metal ions from the environment^8,9^. Additionally, the biomolecules produced during this process act as natural stabilizers for nanoparticles, reducing particle aggregation and increasing their stability^10,11^. Bacterial nanoparticle biosynthesis proceeds via both intracellular and extracellular enzymatic mechanisms. In intracellular pathways, positively charged metal ions are attracted to negatively charged bacterial cell surfaces and subsequently reduced within the cytoplasm or periplasm by oxidoreductase enzymes, such as NADH- or NADPH-dependent reductases, leading to the formation of nanoscale metallic particles. In extracellular synthesis, bacteria secrete reductase enzymes and redox-active biomolecules that facilitate metal ion reduction outside the cell, often accompanied by natural biomolecular capping that enhances nanoparticle stability. Compared to conventional chemical methods, this biogenic approach offers improved environmental compatibility, reduced toxicity, and greater sustainability, making it an efficient strategy for nanomaterial production^12^.

In this context, we selected the acetic acid bacterial strains used in our study because they can be easily obtained from local vinegar production, enabling the development of environmentally friendly, sustainable production systems. Additionally, these bacteria can be used not only for the production of AgNPs but also for bacterial cellulose production^13^. Although there are some studies in the literature on AgNP production from bacterial sources^14–16^, studies on AgNP synthesis by vinegar bacteria are limited, and there is no direct method with this approach yet. This study addresses a knowledge gap in the field and demonstrates the potential of novel biological sources.

Vinegar, a traditional fermentation product, has significant potential for biotechnological applications because of its microbial diversity. Acetic acid bacteria, which play a role in vinegar production, are particularly noteworthy in terms of developing sustainable bioprocesses. Bacterial cellulose production is a biotechnological process carried out by vinegar bacteria such as *Acetobacter xylinum*, and it has been widely investigated for applications in tissue engineering, medicine, and food industries^17,18^.This production process results in a large amount of liquid waste. The aforementioned liquid may contain various organic components and reducing agents, likely possessing the capacity to reduce metal ions.

Given the well-documented antimicrobial, antifungal and antioxidant properties of AgNPs, their integration into polymer matrices or biopolymer films has been proposed as a promising strategy for active and smart food packaging—offering both microbial inhibition and oxidation suppression to extend shelf-life and improve food safety^19^. Moreover, previous studies have demonstrated that composites such as cellulose/AgNP films effectively inhibit common foodborne pathogens (e.g., *Escherichia coli* and *Listeria monocytogenes*) without significantly reducing the viability of human colon cell lines, suggesting acceptable biocompatibility for packaging-contact applications^20^. Compared with plant-mediated nanoparticle synthesis, the use of acetic acid bacteria offers several advantages. These microorganisms produce a variety of extracellular metabolites such as organic acids, enzymes, proteins, and polysaccharides that can act simultaneously as reducing and stabilizing agents during nanoparticle formation. In addition, the use of fermentation-derived liquid waste generated during bacterial cellulose production provides a sustainable and low-cost substrate for nanoparticle synthesis. This approach contributes to waste valorization and aligns with the principles of green chemistry and circular bioeconomy.

The aim of this study is to synthesize silver nanoparticles (AgNPs) using liquid waste generated during the cellulose production of vinegar bacteria, employing an environmentally friendly approach, characterise the structural and morphological properties of these nanoparticles, and evaluate their antimicrobial, antioxidant, and cytotoxic activities to reveal their potential in food packaging applications.

## Results and discussion

### Bacterial supply, AgNP production and characterization

**Fig. 1:**
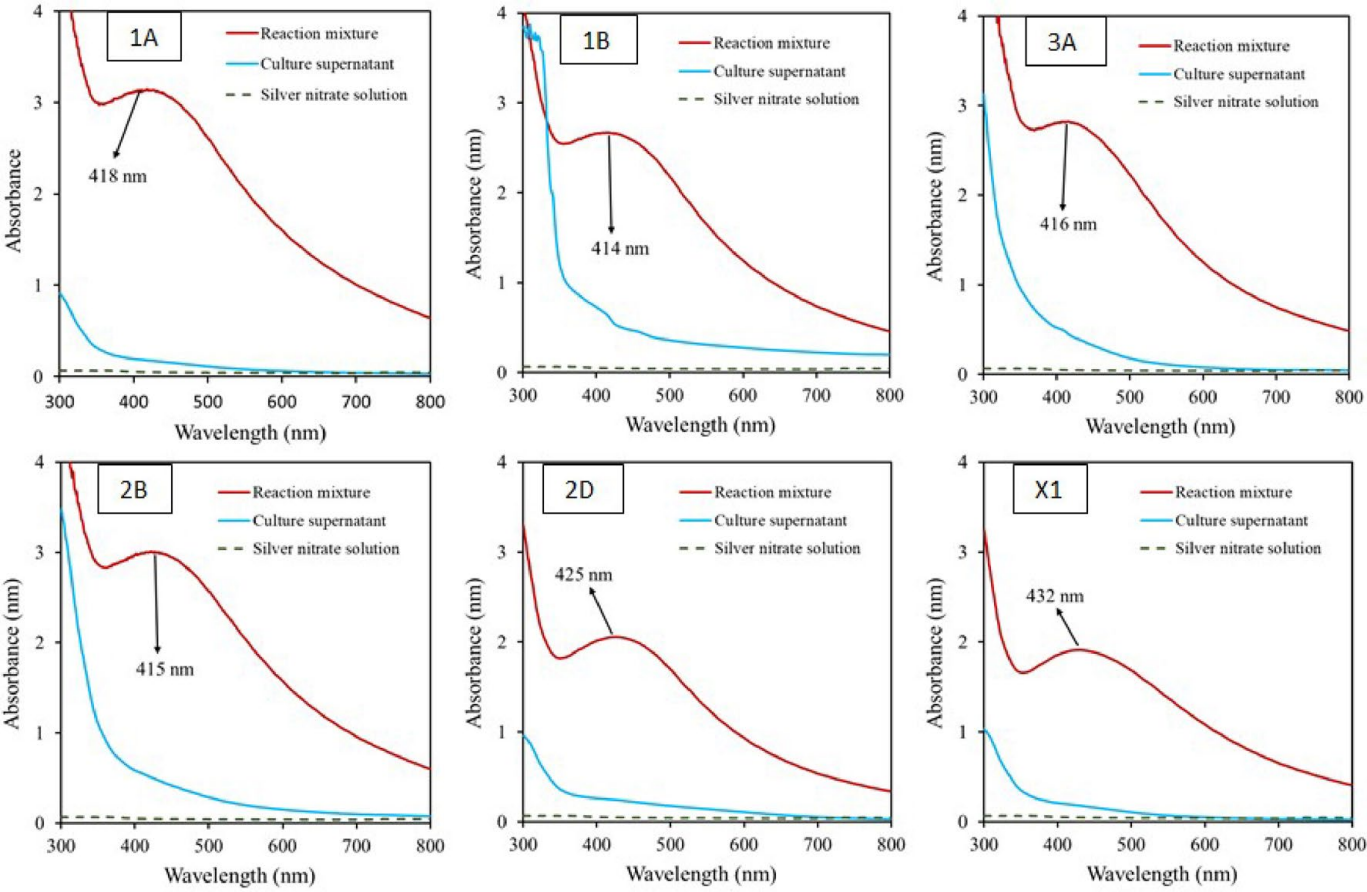
The UV‒Vis spectra of AgNPs synthesized from culture supernatants from isolates 1A, 1B, 3A, 2B, 2D, and X1 are represented by the red solid line. Culture supernatants (blue line) and silver nitrate solution (dashed dark green line) were used as controls.

Among the acetic acid bacteria isolated from homemade vinegars produced in Samsun Province and capable of cellulose synthesis, strains suitable for silver nanoparticle (AgNP) production were identified. The visual change in the reaction mixture from light yellow to reddish brown was the basis for determining suitability, and UV‒Vis spectral analysis verified this shift. Furthermore, the appearance of a distinctive peak in the spectrum representing surface plasmon resonance (SPR) vibration and an absorption peak in the 350–450 nm range confirmed the synthesis of AgNPs. As shown in Fig. 1, AgNPs can be synthesized from the culture supernatants of bacteria isolated from apple vinegar (1A, 1B, 3A), fig vinegar (2B, 2D), and pear vinegar (X1). The physicochemical characteristics of silver nanoparticles (AgNPs) can be inferred from the surface plasmon resonance (SPR) peak. The SPR peak moves to shorter wavelengths as the size of the AgNP decreases. Furthermore, the strength of the SPR peak increases with increasing nanoparticle concentration in the suspension. The width of the absorption bands observed in the SPR spectrum indicates the particle size distribution: wide bands reflect a multisize distribution, whereas narrow bands indicate a homogeneous distribution, i.e., single-size distribution^21,22^. Accordingly, the diameters of the AgNPs synthesized from the culture supernatants of isolates 1A, 1B, 3A, and 2B were smaller, and their distribution was more uniform than those synthesized from isolates 2D and X1. This suggests that the metabolite profiles of these isolates more effectively control the size of AgNPs during the formation process. It was concluded that the concentrations of all synthesized AgNPs were at similar levels. This finding reveals that, despite differences in particle size and distribution, the total nanoparticle synthesis yield was largely preserved among isolates. In addition, the culture supernatant did not exhibit any distinct absorbance peak in the nanoparticle characteristic region, and the AgNO_3_ solution likewise showed no SPR-related absorption. The UV–Vis spectra of AgNPs synthesized using the culture supernatants of isolates, culture supernatant alone and the AgNO_3_ solution are displayed in Fig. 1.

**Fig. 2:**
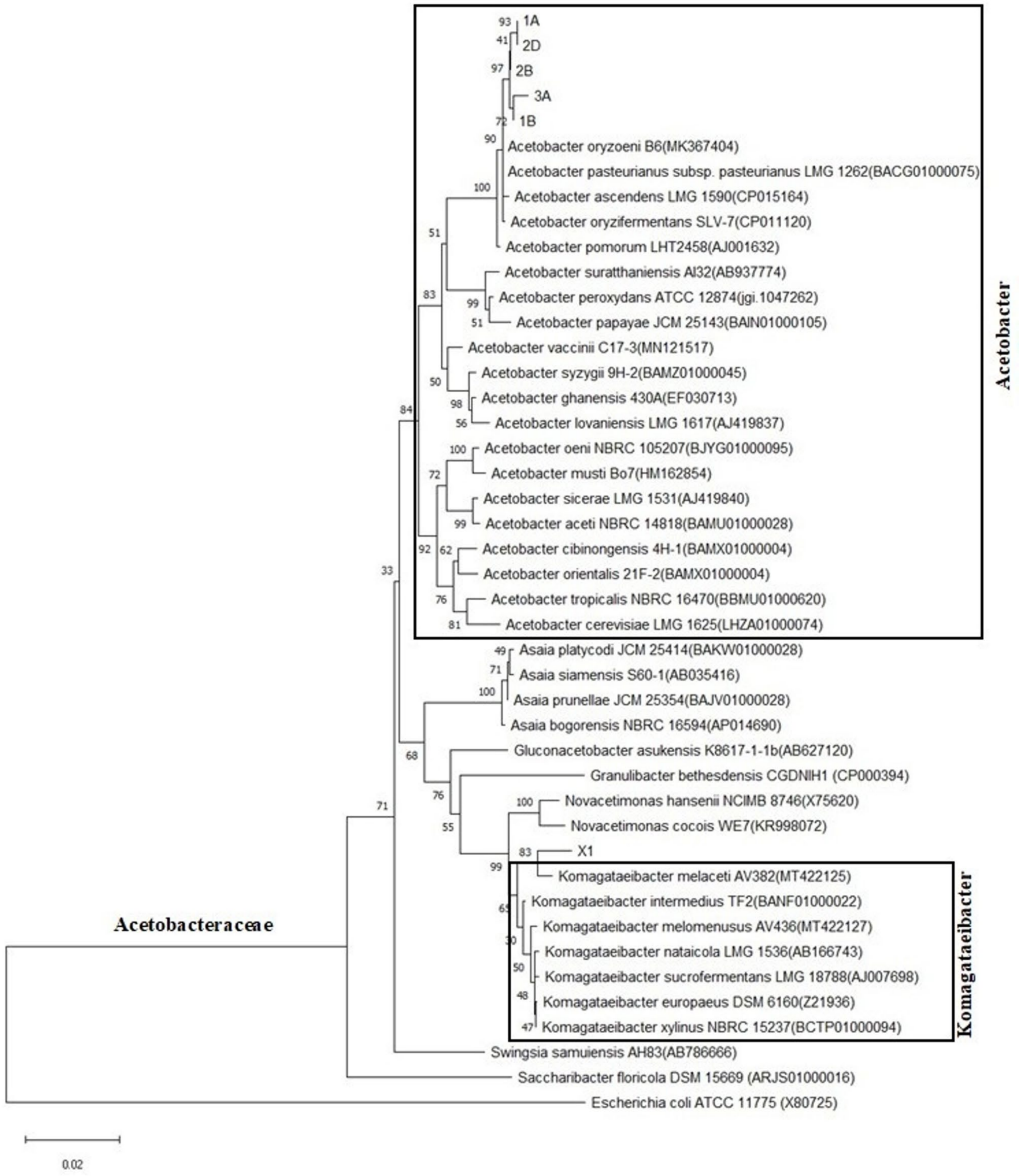
The neighbor‒joining approach was used to create a phylogenetic tree of isolates and closely related species. The percentage of the bootstrap confidence level derived from 1000 iterations is represented by the numbers at the nodes. *E. coli* ATCC 11775, located at the root of the phylogenetic tree, was used as an outgroup in the analysis.

The 16S rRNA gene sequences obtained from the isolates were analyzed and compared with database records using the EzBioCloud platform (Supplementary materials 1–5). The sequences were aligned, and a phylogenetic tree was constructed using the neighbor-joining method (Fig. 2). Accordingly, isolates 1A, 1B, 3A, 2B, and 2D belong to the same monophyletic group with high bootstrap values (93–97%). Additionally, these isolates were closely related to species such as *Acetobacter pasteurianus*, *Acetobacter oryzoeni*, *Acetobacter ascendens*, and *Acetobacter oryzifermentans*. Furthermore, considering their nucleotide similarities, they were determined to be phylogenetically more closely related to *A. pasteurianus* (Supplementary materials 1–5). On the other hand, isolate X1 was found to cluster with species belonging to the *Komagataeibacter* genus and was closely related to *Komagataeibacter medellinensis* and *Komagataeibacter intermedius*. The codes, genera, and accession numbers of the bacteria are displayed in Table 1, which was sourced from the National Centre for Biotechnology Information (NCBI). The accession numbers (PV341489–PV341662) confirm that the sequences were deposited in the NCBI database, ensuring traceability and reproducibility of the taxonomic identification. The presence of both *Acetobacter* and *Komagataeibacter* genera is particularly relevant, as members of these taxa are well known for their bacterial cellulose production capacity, which supports their selection for subsequent nanoparticle synthesis experiments. The phylogenetic tree constructed using the neighbor-joining method showed the evolutionary relationship of the isolates with closely related species (Fig. 2). From the phylogenetic tree, isolates 1A, 1B, 3A, 2B, and 2D are located within a monophyletic group belonging to the genus *Acetobacter* in the family *Acetobacteraceae*, while isolate X1 is located within the *Komagataeibacter* monophyletic group^23^. Furthermore, isolates 1A, 1B, 3A, 2B, and 2D form a clade supported by high bootstrap values (93–97%). This clustering suggests that the isolates likely belong to the same taxonomic group. However, due to the phenotypic similarities among acetic acid bacteria, differentiation at the species level is generally difficult. The results indicate that a chemotaxonomic characterization, which includes genotypic characteristics, is necessary to achieve taxonomic certainty for species-level differentiation.

**Table 1:** Bacterial codes, genera, and accession numbers obtained from NCBI.

Bacteria code	Isolated source	Bacteria species	Accession number
1A	Apple Cider Vinegar	*Acetobacter* sp.	PV341489
1B	Apple Cider Vinegar	*Acetobacter* sp.	PV341624
2B	Fig Vinegar	*Acetobacte*r sp.	PV341496
2D	Fig Vinegar	*Acetobacter* sp.	PV341505
3A	Apple Cider Vinegar	*Acetobacter* sp.	PV341506
X1	Pear Vinegar	*Komagataeibacter* sp.	PV341662

### Physicochemical characterization of the produced AgNPs

#### Microscopic characterization of the AgNP (electron microscope) results

**Fig. 3:**
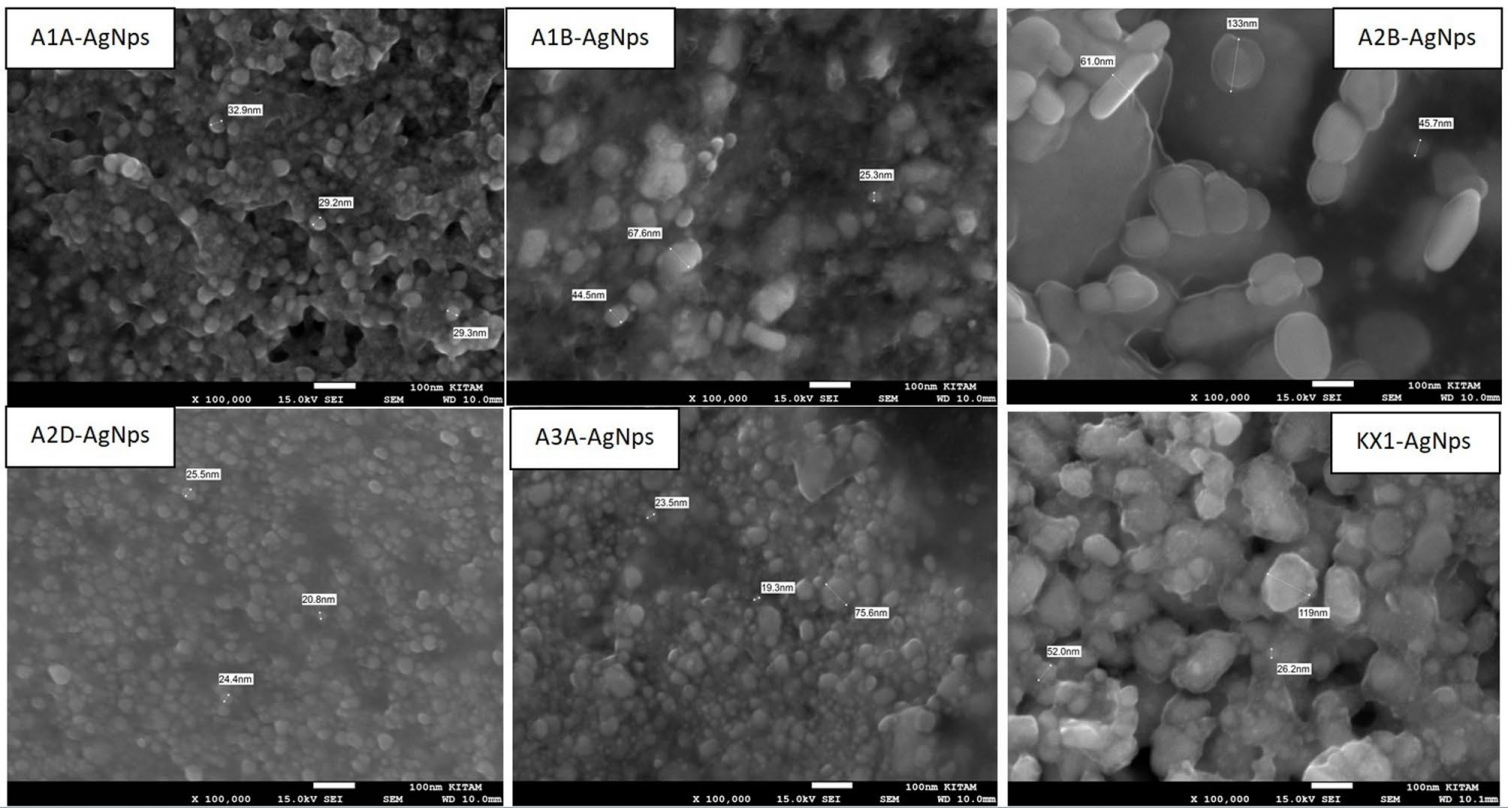
SEM images of the surfaces of the AgNPs.

Figure 3 shows SEM images of AgNPs synthesized from culture supernatants of six bacterial strains isolated from different vinegar sources. SEM investigations revealed strain-specific differences in the size and distribution of the synthesized nanoparticles, confirming that AgNPs can be produced from culture supernatants of all the isolates. AgNPs with diameters of 20–40 nm, 30–60 nm, and 30–70 nm were synthesized from the culture supernatants of *Acetobacter* sp. 1B, 2D, and 3A (A1B-AgNPs, A2D-AgNPs, and A3A-AgNPs), respectively, and exhibited a more homogeneous distribution than the other synthesized nanoparticles did. The AgNPs synthesized from *Acetobacter* sp. 1A and *Komagataeibacter* sp. X1 culture supernatants (A1A-AgNPs and KX1-AgNPs) were spherical, with sizes ranging from 30 to 100 nm, although they had relatively irregular morphologies. On the other hand, AgNPs synthesized with *Acetobacter* sp. 2B culture supernatant (A2B-AgNPs) exhibited rod-like shapes and were agglomerated.

The EDS map in Table 2 shows that the structure of the AgNP has the same components. Ag is present in a proportion of 46–93%; this is to be expected, as it is the main component of silver nanoparticles. Carbon, nitrogen, and oxygen are present up to 20% by mass each. The presence of organic compounds on the surface likely indicates the presence of reducing/coating agents. P, S, and Cl (< 4%) are contributions from biomolecules or the synthesis environment. Na and Al are present in trace amounts and are thought to be contaminants or buffer salts. Ahmed et al. (2016) reported that in plant-based syntheses, high ratios of C, N, and O were observed alongside Ag via EDS^2^. Bar et al.^24^ reported that phenolic coatings around silver nanoparticles appear as C and O in EDS. According to Durán et al.^25^, elemental analysis of protein-capped silver nanoparticles revealed the presence of nitrogen and phosphorus signals characteristic of the protein layer around AgNPs. SEM images of the AgNP surfaces revealed that the densest and brightest nanoparticle distribution was observed in the A3A-AgNP sample. The EDS map reveals that the particles are nearly entirely composed of silver.

**Table 2:** EDS mapping results of the AgNPs (wt%).

Element (%)	A1A-AgNPs	A1B-AgNPs	A2B-AgNPs	A2D-AgNPs	A3A-AgNPs	KX1-AgNPs
C	15.48	18.69	14.21	18.99	3.98	22.48
N	10.58	11.70	8.09	13.72	–	12.09
O	8.17	13.03	6.78	11.76	1.35	12.28
Na	–	–	–	0.20	–	1.26
Al	–	–	–	–	–	0.15
P	2.01	1.84	1.85	1.92	0.26	1.78
S	1.08	0.88	1.05	0.63	0.68	0.91
Cl	3.67	3.34	3.31	1.85	–	2.24
Ag	59.01	50.52	64.71	50.93	93.74	46.80

#### Fourier transform infrared spectroscopy (ATR-FTIR) results for AgNPs

**Fig. 4:**
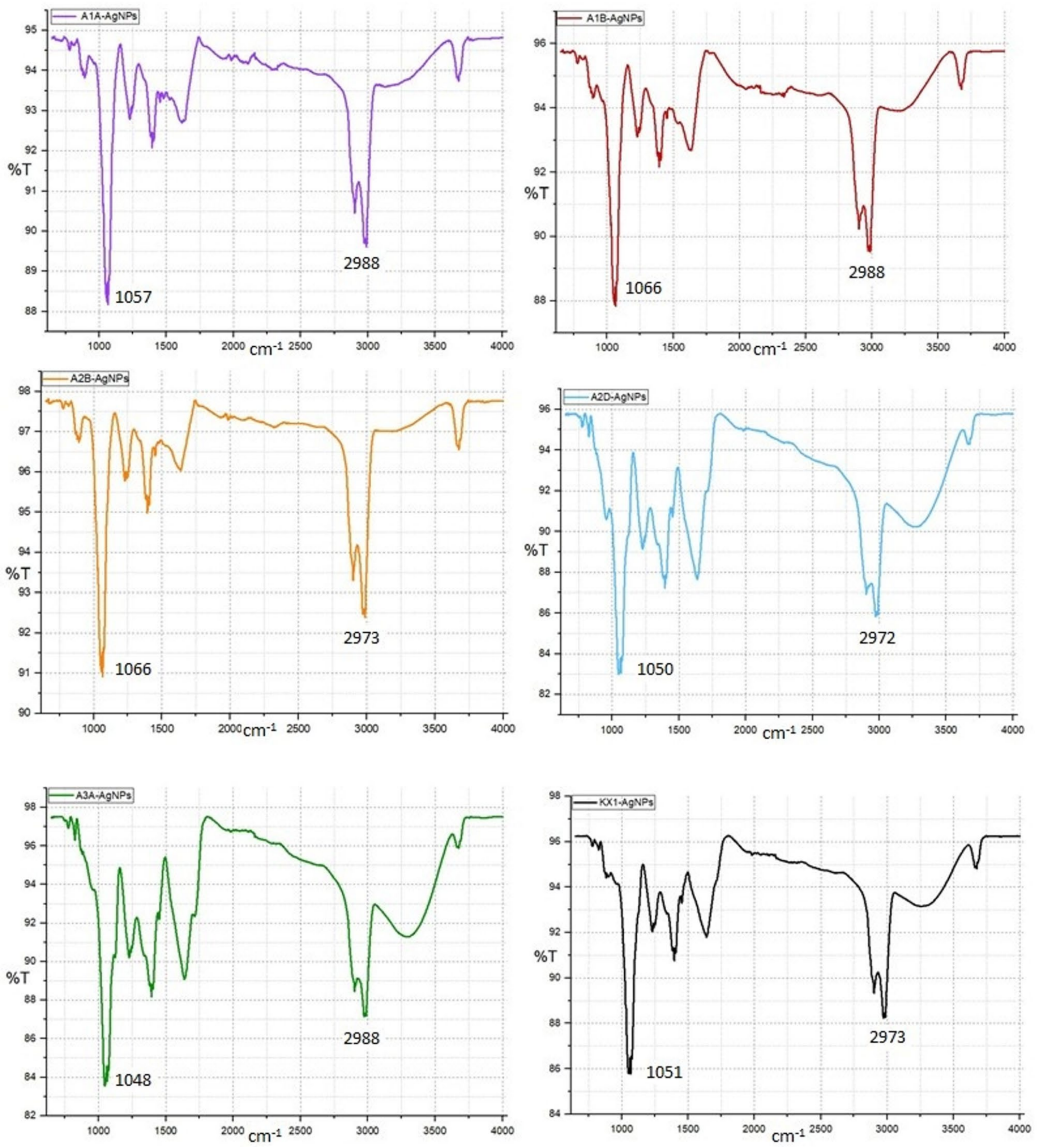
ATR-FTIR spectra of the AgNPs.

The FTIR spectra of silver nanoparticles (AgNPs) can exhibit various peaks due to stabilizers adsorbed on the surface, biological coating agents (plant extracts, proteins, polysaccharides, and polymers), and functional groups in the synthesis environment. 2980–2990 cm^−1^: C–H (alkane): This range denotes the presence of an aliphatic structure and correlates to the asymmetric stretching vibrations of acyclic methyl (–CH_3_) and methylene (–CH₂–) groups. This indicates the presence of organic residues (e.g., CH_3_ and CH₂ groups within phenolic structures) on the surface of the AgNPs. 2904–2899 cm^−1^: C–H (alkane): These are also symmetric stretching vibrations. Thus, these regions support the presence of saturated hydrocarbon chains in the compound^26^. 1672–1585 cm^−1^: This range may be critical for several different bonds: C = C double bonds (aromatic or alkene), N–H bending (secondary amine), or C = N bonds. This could also be a slightly shifted C = O bond. The presence of protein/enzyme coatings utilized in AgNP production is indicated by this region^27^. 1393–1395 cm^−1^: This finding supports the presence of CH_3_ bending vibrations and alkane chains or alkyl groups, indicating the presence of biomolecules (proteins, amino acids) on the AgNP surface. 1232–1226 cm^−1^: This region typically indicates C–O–C (ether), ester (C–O), or phenolic C–O bonds. There is an oxygen-containing group, which could be an alcohol, ester, or ether. This peak is associated with plant polysaccharides or phenolic compounds (e.g., gallic acid) involved in the stabilization of AgNPs^28^. 1077–1043 cm^−1^: C–O stretching vibrations (alcohols, ethers, carboxylic acid derivatives). The C–N bond may also be observed in this region, indicating the presence of an oxygen- or nitrogen-containing functional group. This region is often associated with sugar derivatives (glucose, cellulose), flavonoids, and protein side chains. The ATR-FTIR spectra of the AgNPs are displayed in Fig. 4. Characteristic surface groups such as –OH, C = O, and C-N are present in every sample. These groups enable the nanoparticles to interact electrostatically and chemically with the cell walls of microorganisms^29^. The KX1-AgNP and A3A-AgNP spectra have the richest number of functional groups, which is a factor that supports their biological activity^28^. The surface groups of A2B-AgNPs are also quite active, which explains their antimicrobial effects *on P. aeruginosa* and *Y. enterocolitica*.

#### XRD results of the AgNPs

**Fig. 5:**
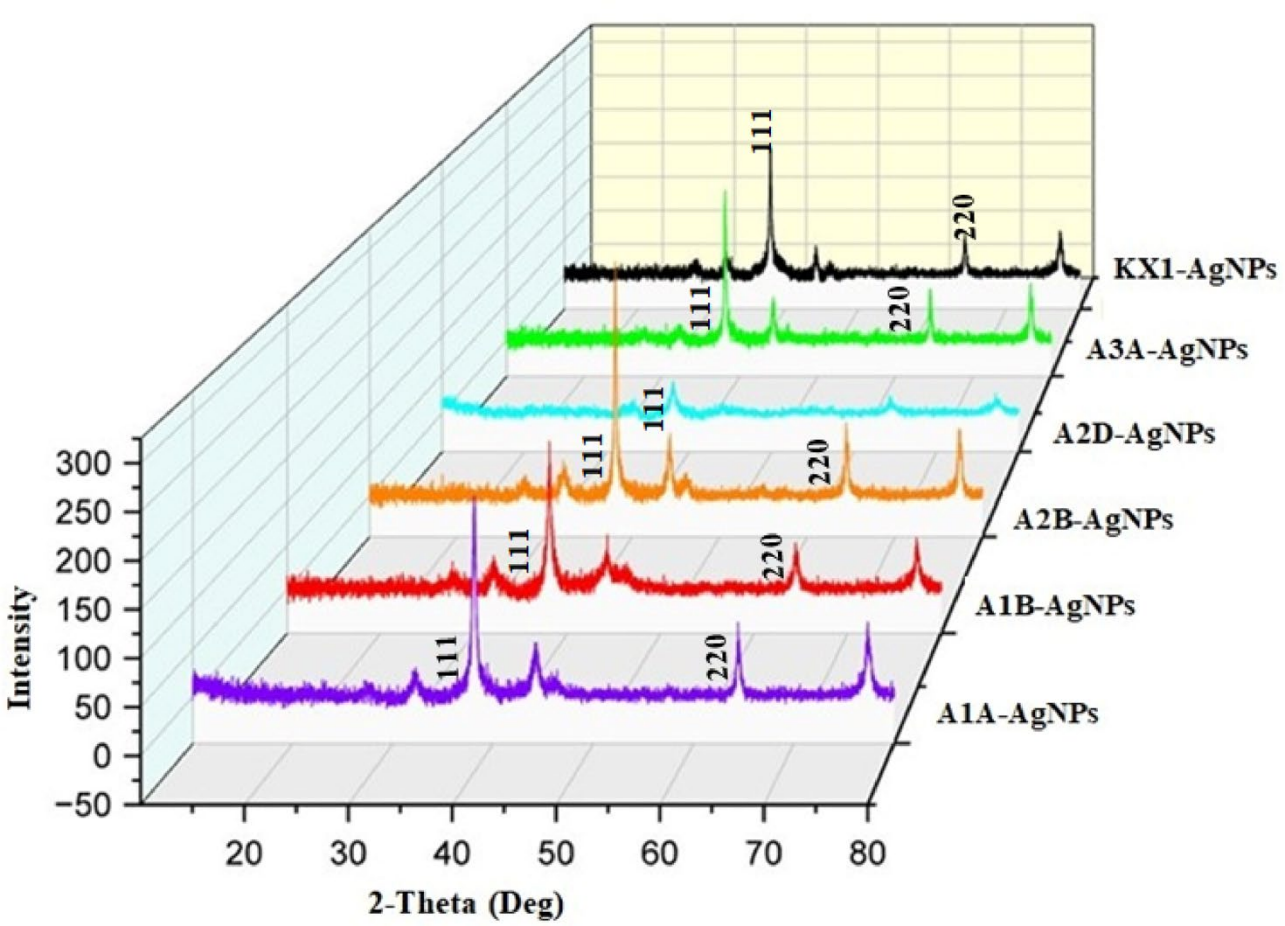
XRD characterization results for the AgNPs.

The XRD results demonstrated that the crystal structure of the AgNPs was maintained. The gel film XRD patterns showed several diffraction peaks at 2θ values of approximately 38.1°, 44.2°, 64.4°, and 77.3°. The typical face-centered cubic (FCC) crystal structure of silver (Ag) is represented by these 2θ angles. The sharp and intense XRD patterns of the films suggest a high probability of the presence of morphologically round and polyhedral AgNPs. The XRD patterns are similar. The observed XRD peaks indicate silver nanoparticles with high crystallinity. The dominance of the (111) plane suggests that AgNPs are more prone to crystallization in this direction, which is related to surface energy. Shankar et al.^30^ demonstrated that XRD analysis of biologically synthesized AgNPs confirmed the FCC structure and that the (111) plane was the dominant peak. The absence of any phase impurities between the peaks indicates that the synthesized AgNPs have high phase purity. This structure is an important indicator that directly affects the functionality of AgNPs in optical, catalytic, and biomedical applications. Silver nanoparticles crystallized in an FCC structure with distinctive XRD peaks that matched the (111), (200), (220), and (311) planes, according to Ali et al.,^31^. Figure 5 displays the AgNP XRD characterization results.

#### Dynamic light scattering (DLS)

**Fig. 6:**
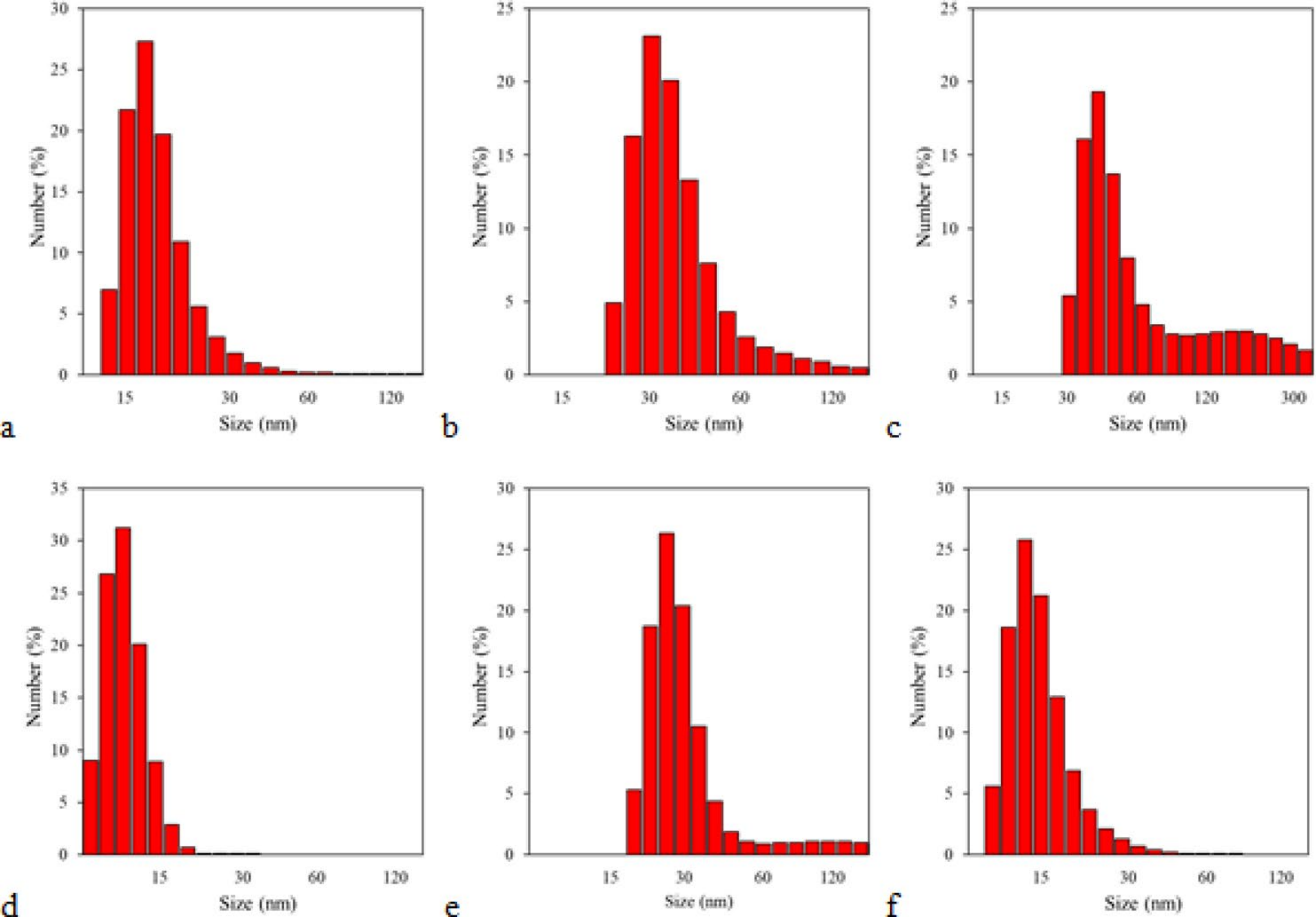
Particle size distribution of A1A-AgNPs (**a**), A1B-AgNPs (**b**), A2B-AgNPs (**c**), A2D-AgNPs (**d**), A3A-AgNPs (e**)**, and KX1-AgNPs (**f**) on the basis of number.

Dynamic light scattering (DLS) is a widely used technique for determining the hydrodynamic diameter (Z-average) and size distribution (Polydispersity Index: PDI) of nanoparticles in colloidal solutions by analyzing fluctuations in scattered light arising from their Brownian motion. They are crucial to the industrial and clinical applications of nanoparticles^32^. Data from DLS analysis showed that there were notable differences in the particle distribution patterns among the synthesized nanoparticles (Fig. 6). The PDI value indicates the width of the particle size distribution. Nanoparticles with PDI values < 0.3 and 0.3–0.5 are considered to be strongly and moderately monodisperse, respectively, while those with PDI values above 0.5–1 are considered to be polydisperse in aqueous solutions (from broader distributions to highly varied distributions)^33,34^. A1A-AgNPs (Z-average: 85.05 nm; PDI: 0.318; Intercept: 0.849) and KX1-AgNPs (85.18 nm; PDI: 0.339; Intercept: 0.860) exhibited similar hydrodynamic diameters with moderately monodisperse distributions. A1B-AgNPs (Z-average: 97.41 nm; PDI: 0.402; Intercept: 0.849) and A3A-AgNPs (Z-average: 179.1 nm; PDI: 0.401; Intercept: 0.842) showed higher PDI values, indicating broader and more heterogeneous size distributions. In the case of A2B-AgNPs (Z-average: 169 nm; PDI: 0.274; Intercept: 0.870), the presence of two distinct peaks (49.39 nm—74.2% and 203.1 nm—25.8%) demonstrated a bimodal distribution, suggesting partial aggregation or the coexistence of two particle populations. Further, the elevated Z-average value for A2B-AgNPs confirms the influence of the larger fraction on the overall mean size. In contrast, A2D-AgNPs exhibited a Z-average of 90.14 nm with a PDI of 0.291, indicating a relatively narrower distribution and a more homogeneous nanoparticle. The results also revealed a significant disparity between the particle sizes determined by SEM and the hydrodynamic diameters measured by DLS. This is due to the disproportionate effect that even a small number of larger nanoparticles can have on the measurement result in DLS analysis, and the method’s assumption that the particles are spherical^35^.

#### Zeta potential (ζ)

**Fig. 7:**
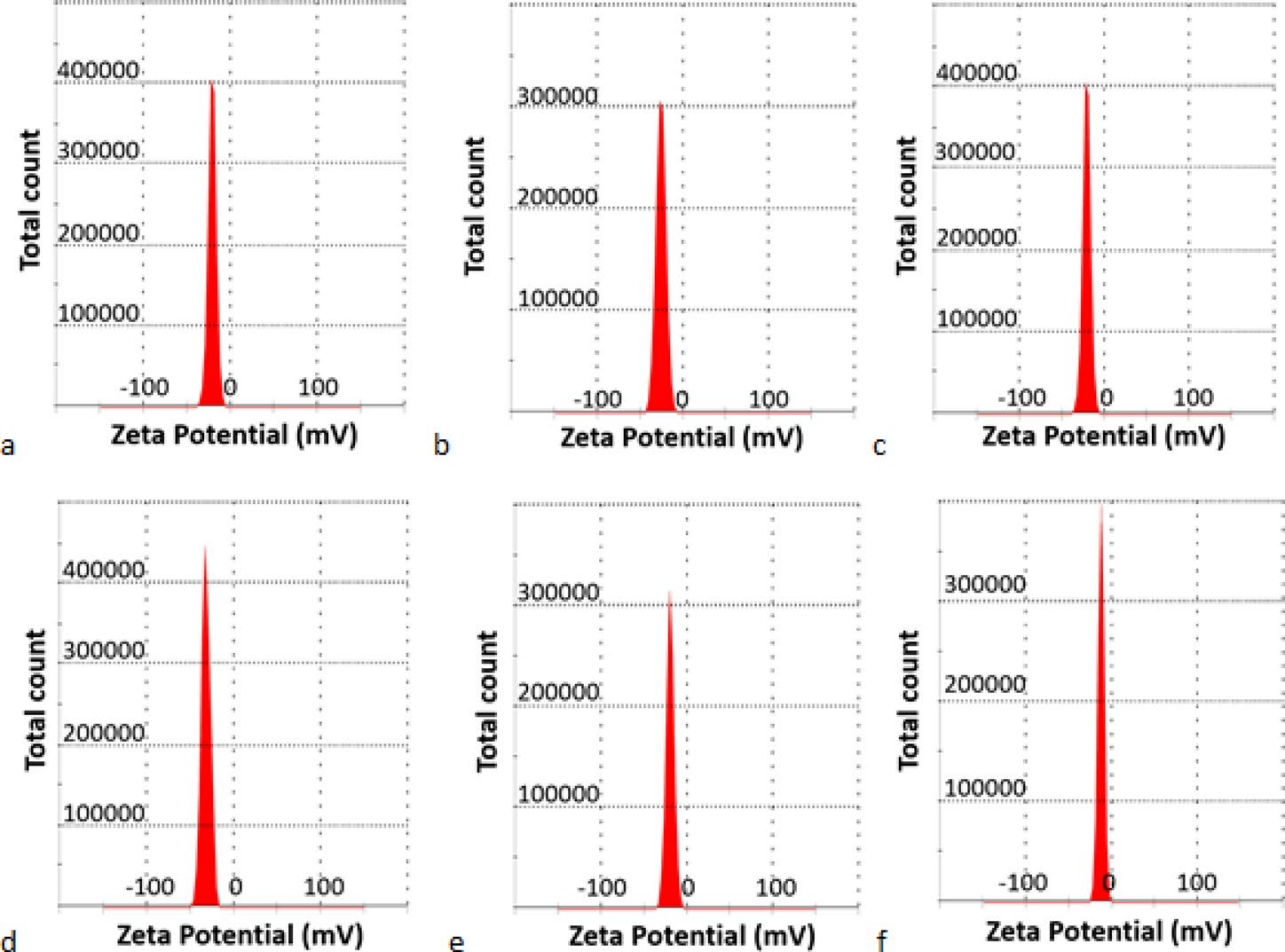
Zeta potential of A1A-AgNPs (**a**), A1B-AgNPs (**b**), A2B-AgNPs (**c**), A2D-AgNPs (**d**), A3A-AgNPs (**e**), and KX1-AgNPs (**f**) solutions at 25 °C.

Another critical factor affecting the efficacy of nanoparticles in clinical and biotechnological applications is the zeta potential value, which allows us to determine the surface charge and evaluate its electrostatic stability of the nanoparticles (Ghidoni et al., 2025). Nanoparticles with zeta potentials >  ± 30 mV and in the range of ± 10 – ± 30 mV exhibit high and moderate colloidal stability, respectively, while those with zeta potentials between − 10 mV and + 10 mV tend to aggregate. Zeta potential measurements showed that all synthesized AgNPs possess negatively charged surfaces with values ranging from − 12.3 to − 31.8 mV (Fig. 7). It indicates the presence of anionic functional groups adsorbed to the nanoparticle surface, likely originating from reducing and/or stabilizing agents used during synthesis, such as peptides, phenolic compounds, and polysaccharides^36^. Among the tested nanoparticles, A2D-AgNPs exhibited the highest absolute zeta potential (− 31.8 mV), followed by A1B-AgNPs (− 25.5 mV), A1A-AgNPs (− 20.7 mV), A3A-AgNPs (− 19.7 mV), A2B-AgNPs (− 18.8 mV), and KX1-AgNPs (− 12.3 mV). From a colloidal stability perspective, A2D-AgNPs can be classified as highly stable, as zeta potential values with an absolute magnitude ≥ 30 mV are generally considered indicative of strong electrostatic repulsion. A1B-AgNPs, A1A- AgNPs, A3A- AgNPs, and A2B-AgNPs showed moderate stability. In contrast, KX1-AgNPs exhibited the lowest absolute zeta potential, suggesting relatively weak electrostatic stabilization and a higher susceptibility to aggregation over time. The observed differences in zeta potential among nanoparticles reflect variations in the chemical composition and capping efficiency of culture supernatants^37^.

### Antimicrobial activity of AgNPs

The inhibition zone diameter indicates the overall antimicrobial effect of AgNPs on microorganisms. The greatest effect is generally considered to be ≥ 12 mm. *Y. enterocolitica* (12.5–13.6 mm, generally highly potent), *B. subtilis* (12.7–14.9 mm, particularly effective A1A-AgNP and A4B-AgNP), *S. aureus* (11.2–12.6 mm) and *A. baumannii* (10.2–13.5 mm, effective despite being resistant) demonstrated strong antimicrobial effects. No antimicrobial activity was detected in the negative control.

Compared with Gram-positive bacteria, Gram-negative bacteria are more susceptible to the effects of AgNPs. Gram-negative bacteria are more susceptible to AgNPs because their thin peptidoglycan layer facilitates nanoparticle penetration^38^. However, in this study, a significant effect was also observed in gram-positive species such as *B. subtilis*, indicating the effects of particle size and coating agent.

When analyzed (Table 3), both AgNPs and streptomycin strongly inhibited pathogens such as *Y. enterocolitica*, *B. subtilis*, and *C. albicans*. AgNPs were clearly almost ineffective (all zones resistant or very low) against *E. faecalis*, whereas streptomycin generally produced larger zones of inhibition and was ineffective against resistant strains such as ESBL *E. coli* and MRSA. The results of the MIC and MLC studies (concentrations: 500-250-125-62-32-16 µg/mL) are shown in Table 4.

**Table 3:** Antimicrobial activity results of AgNP against the test bacteria.

Test microorganisms	A1A-AgNPs	A1B-AgNPs	A2B-AgNPs	A2D-AgNPs	A3A-AgNPs	KX1-AgNPs	Streptomycin 10 µg
*E. faecalis*	R	8.3 ± 0.4ᵇ	9.1 ± 0.3ᵇ	10.6 ± 0.5ᵃ	10.4 ± 0.4ᵃ	8.8 ± 0.3ᵇ	R
*K. pneumoniae*	10.8 ± 0.5ᵃ	10.2 ± 0.4ᵃ	8.9 ± 0.3ᵇ	9.2 ± 0.4ᵇ	7.5 ± 0.3ᶜ	8.8 ± 0.4ᵇ	R
*A. baumannii*	11.3 ± 0.6ᵇ	13.5 ± 0.5ᵃ	12.9 ± 0.4ᵃ	10.5 ± 0.3ᵇ	10.2 ± 0.4ᵇ	9.0 ± 0.3ᶜ	R
*B. cereus*	9.0 ± 0.3ᵇ	9.1 ± 0.4ᵇ	9.4 ± 0.3ᵇ	9.4 ± 0.3ᵇ	9.3 ± 0.3ᵇ	10.4 ± 0.4ᵃ	23.1 ± 0.8
*B. subtilis*	14.9 ± 0.6ᵃ	12.8 ± 0.5ᵇ	12.7 ± 0.4ᵇ	11.0 ± 0.4ᶜ	11.4 ± 0.5ᶜ	9.7 ± 0.3ᵈ	24.9 ± 0.9
*Y. enterocolitica*	11.4 ± 0.5ᵇ	12.2 ± 0.4ᵇ	12.5 ± 0.5ᵇ	13.6 ± 0.6ᵃ	13.2 ± 0.5ᵃ	12.5 ± 0.4ᵇ	26.1 ± 1.0
*P. aeruginosa*	11.8 ± 0.4ᵇ	11.6 ± 0.5ᵇ	10.4 ± 0.4ᶜ	12.7 ± 0.5ᵃ	10.8 ± 0.4ᶜ	11.0 ± 0.4ᶜ	14.8 ± 0.7
*C. albicans*	11.8 ± 0.5ᵇ	9.9 ± 0.4ᶜ	13.2 ± 0.6ᵃ	10.7 ± 0.4ᵇ	11.1 ± 0.5ᵇ	11.4 ± 0.4ᵇ	25.1 ± 0.9
*S. aureus*	11.8 ± 0.5ᵇ	11.2 ± 0.4ᵇ	12.6 ± 0.5ᵃ	8.8 ± 0.3ᶜ	8.2 ± 0.3ᶜ	8.6 ± 0.3ᶜ	24.2 ± 0.8
MRSA	10.0 ± 0.4ᵇ	10.1 ± 0.4ᵇ	10.5 ± 0.4ᵇ	11.6 ± 0.5ᵃ	11.1 ± 0.4ᵃ	11.7 ± 0.5ᵃ	R
*E. coli*	8.1 ± 0.3ᶜ	9.9 ± 0.4ᵇ	10.1 ± 0.4ᵇ	11.8 ± 0.5ᵃ	10.8 ± 0.4ᵇ	9.6 ± 0.3ᵇ	10.4 ± 0.5
ESBL *E. coli*	8.8 ± 0.3ᶜ	9.2 ± 0.4ᵇ	10.7 ± 0.4ᵃ	11.5 ± 0.5ᵃ	10.0 ± 0.4ᵇ	11.3 ± 0.5ᵃ	R

The values are expressed as mean ± standard deviation (n = 3). Different superscript letters in the same row indicate statistically significant differences (*p* < 0.05). Abbreviation: R, Resistant.

**Table 4:** MIC and MLCs (µg/mL) of AgNPs for test microorganisms.

Test micrororganisms	A1A-AgNP		A1B-AgNP		A2B-AgNP		A2D-AgNP		A3A-AgNP		KX1-AgNP	
	MIC	MLC	MIC	MLC	MIC	MLC	MIC	MLC	MIC	MLC	MIC	MLC
*E. faecalis*	–	–	–	–	–	–	–	–	–	–	–	–
*K. pneumoniae*	–	–	500	500	–	–	250	500	–	–	250	–
*A. baumannii*	500	–	–	–	500	500	–	–	–	–	–	–
*B. cereus*	–	–	32	500	–		–	–	–	–	–	–
*B. subtilis*	–	–	–		–	–	–	–	–	–	–	–
*Y. enterocolitica*	500	–	62	500	500	500	–	–	–	–	–	–
*P. aeruginosa*	–	–	250	250	–		–	–	–	–	–	–
*C. albicans*	–	–	–		–	–	–	–	–	–	–	–
*S. aureus*	500	500	500	–	–	–	16	16	–	–	–	–
MRSA	500	500	250	250	–		–	–	–	–	250	250
*E. coli*	–	–	500	–	–		250	250	500	–	–	–
ESBL* E. coli*	500	–	500	–	–	–	–	–	–	–	250	250

MIC and MLC values are expressed as µg/mL. MIC represents the lowest concentration inhibiting visible growth, while MLC indicates the lowest concentration resulting in no microbial growth on agar plates.

MIC data indicate the concentration at which bacteria cannot multiply in the presence of AgNPs. A low MIC value (e.g., ≤ 62 µg/mL) indicates high antimicrobial activity. ESBL *E. coli* (A1C-AgNP): An effective concentration of 16 µg/mL indicates high sensitivity despite bacterial resistance. *S. aureus* (A2D-AgNP): A concentration of 16 µg/mL indicates an effective AgNP formulation. *B. cereus* (A1B-AgNP) was effective at 32 µg/mL, indicating the presence of antimicrobial activity*.* High MIC values of 500 µg/mL are typically observed in resistant bacteria such as MRSA, *K. pneumoniae*, and *A. baumannii*.

The small, uniformly distributed spherical nanoparticles in A1A-AgNPs and A1B-AgNPs offer advantages in terms of stability and surface interaction. However, their crystallinity is low, which may limit their antimicrobial activity^29,39^. Agglomeration was observed in A2B-AgNPs. This situation may negatively affect biological activity by reducing the surface area^4^. The spherical morphology of the A2D-AgNPs and A3A-AgNPs is preserved, and the particles are dense and relatively well dispersed. This characteristic may explain the high efficacy of the A3A-AgNPs against *S. aureus*. When the XRD patterns were examined, the KX1-AgNP sample presented the sharpest and most distinct peak, indicating high crystallinity. This characteristic may improve particle stability and reactivity, potentially contributing to antibacterial activity^4^. Moderate crystallinity is observed in A3A-AgNPs and A2D-AgNPs. The high efficacy of A3A-AgNPs against *S. aureus* is directly related to this structure. A1A-AgNPs and A1B-AgNPs have relatively less crystalline structures and exhibit lower antimicrobial effects. In their studies, Marambio-Jones and Hoek^40^ noted that the MIC values of AgNPs typically ranged from 25 to 100 µg/mL. The values obtained in this context, such as 16–32 µg/mL, indicate that AgNPs exhibit high bioeffectiveness. Taken together, these observations indicate a clear relationship between nanoparticle physicochemical characteristics and antimicrobial performance. Overall, the antimicrobial results indicate that the synthesized AgNPs exhibited variable levels of activity depending on the tested microorganism and the assay method. In the agar diffusion assay, KX1-AgNPs produced measurable inhibition zones against several microorganisms, including MRSA and ESBL-positive E. coli, indicating an inhibitory effect under agar-based conditions. However, MIC and MLC results suggest that the bactericidal activity was limited and observed only for certain strains at relatively high concentrations. The discrepancy between inhibition zone diameters and MIC values may be related to the diffusion of Ag⁺ ions released from the nanoparticles in the agar medium. Therefore, the antimicrobial activity of KX1-AgNPs should be considered moderate and strain-dependent.

MLC indicates the concentration at which bactericidal effects are observed. Compared with the MIC, the concentration of the substance is generally slightly greater. MLC values of 16 µg/mL were observed for *S. aureus* (A2D-AgNP) and 250 µg/mL for MRSA in A1B-AgNP and KX1-AgNP. Many studies have shown that some AgNP formulations have bacteriostatic effects and exhibit bactericidal properties. The results of Shrivastava et al. 2017 are consistent with the 16–250 µg/mL range reported in this study^41^. The observed activity against resistant strains such as MRSA and ESBL-positive *E. coli* suggests selective antibacterial potential. The direct breakdown of the cell membrane caused by the binding of Ag ions to sulfur-containing proteins in bacteria could be the mechanism of action for the antibacterial properties of Ag/Fe_2_O_3_-NCs. Free radicals may produce oxidative stress, which can subsequently cause the cell membrane to contract and separate from the cell wall, ultimately resulting in cell death^42,43^. These findings suggest that AgNPs AgNPs may represent promising adjunct antimicrobial materials, particularly against resistant strains.

### Antioxidant activity of the AgNPs

**Fig. 8:**
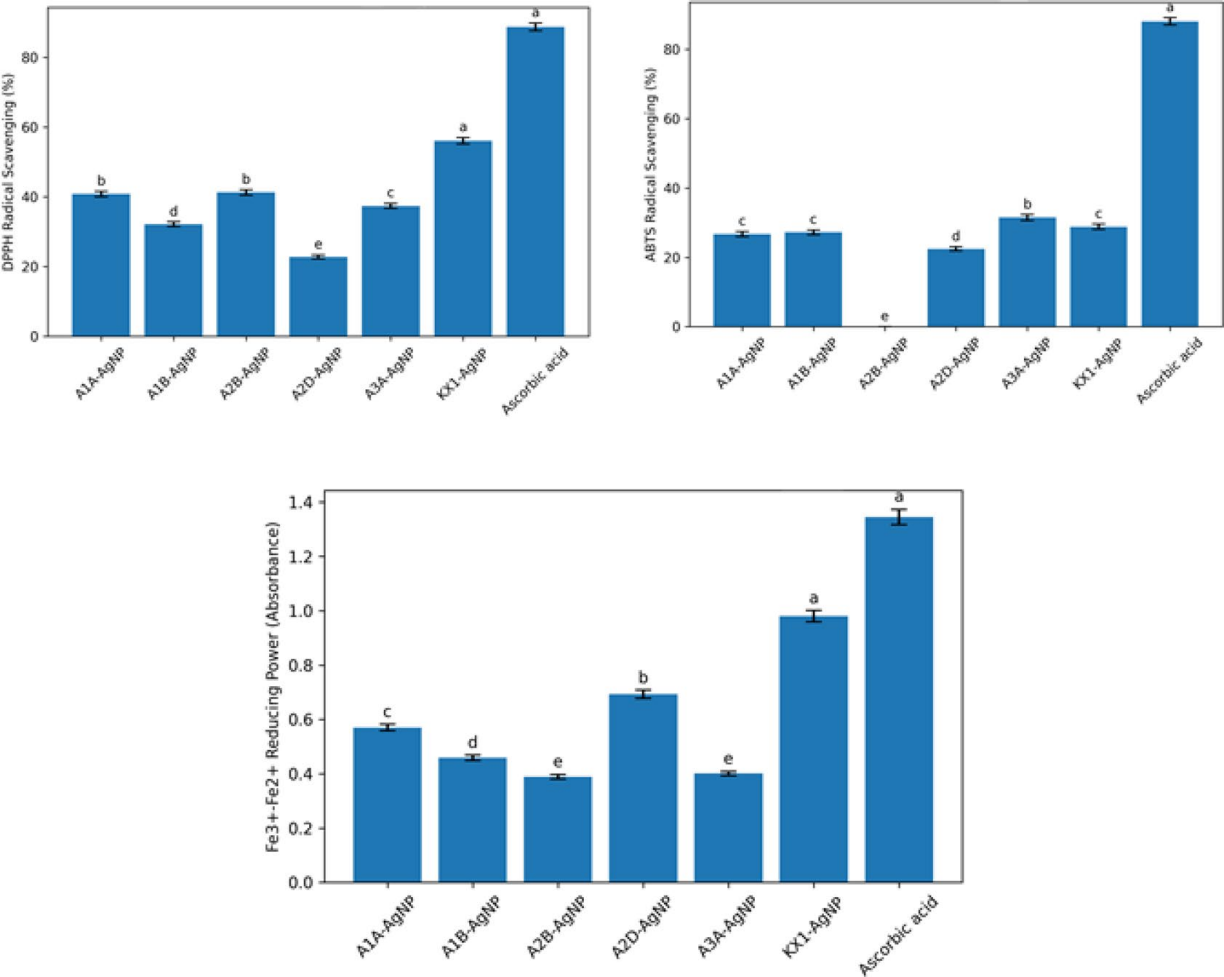
DPPH/ ABTS radical scavenging activity, and Fe^3^⁺–Fe^2^⁺ reducing power of AgNPs and ascorbic acid (positive control) at 800 µg/mL. Values are expressed as mean ± SD (n = 3). Different letters indicate statistically significant differences (*p* < 0.05).

Silver nanoparticles synthesized via the green synthesis method with acetic acid bacteria (*Acetobacter* sp., *Komagataeibacter* sp.) isolated from homemade vinegars were evaluated. The AgNPs (A1A-AgNP, A1B-AgNP, A2B-AgNP, A2D-AgNP, A3A-AgNP, and KX1-AgNP) at different concentrations (50–800 µg/mL) were evaluated via DPPH, ABTS, and iron (Fe^3^⁺) reduction tests, with ascorbic acid used as the reference substance. The results obtained are presented in Fig. 8.

Owing to their special physicochemical characteristics, including a high surface area/volume ratio and unique redox potentials, AgNPs have a substantial impact on a range of biological functions and help to effectively neutralize free radicals^44^. The results of the DPPH free radical scavenging assay demonstrated that all AgNP samples exhibited substantial increases in inhibition values as their concentration increased. At a concentration of 800 μg/mL, KX1-AgNPs had the highest inhibitory value (inhibition rate: 56.01%). These results suggest that KX1-AgNPs have unique antioxidant qualities and greater potential to scavenge DPPH radicals. These values, however, are less than those of ascorbic acid, which was employed as a positive control and produced an 88.65% inhibition rate. This finding shows that AgNPs have a limited antioxidant ability compared with ascorbic acid, although they show notable activity in certain samples.

ABTS research revealed a concentration-dependent increase in the inhibitory activity of the AgNPs (Fig. 8). This test is an effective method for evaluating both hydrophilic and lipophilic antioxidants and reflects the radical scavenging capacity of nanoparticles in aqueous media. The highest inhibition percentages were observed for samples A3A-AgNP (31.48%), KX1-AgNP (28.79%), and A1B-AgNP (27.21%) (Fig. 8). However, A2B-AgNPs had 0.00% inhibitory effects at all concentrations, which suggests that the capacity of A2B-AgNPs to remove ABTS radicals is negligible. Ascorbic acid again exhibited the highest activity, with 88.05% inhibition. According to the results of the present study, the KX1-AgNP sample exhibited strong antibacterial activity against multidrug-resistant bacteria such as *K. pneumoniae*, MRSA, and ESBL-positive *E. coli*, whereas the A3A-AgNP and KX1-AgNP samples presented notable activity in the DPPH and ABTS antioxidant tests. Acharya et al. (2025) concluded that nanoparticles produced by green synthesis have high antioxidant and antimicrobial potential, which is consistent with the literature supporting Iravani’s (2011) critical role of biomolecules in these functions^4,45^. Thus, the obtained data once again confirm the potential of using the produced nanoparticles as both antioxidant and antimicrobial agents.

When the reduction capacities of AgNPs against Fe⁺^3^ ions were evaluated, the highest absorbance values were observed for KX1-AgNP (0.980), A2D-AgNP (0.693), and A1A-AgNP (0.570). The fact that KX1-AgNP also stands out in the reduction test against Fe⁺^3^ ions demonstrates the high electron donation capacity on the surface and the effectiveness of AgNPs in redox reactions. Another notable result is the high reduction capacity of the A2D-AgNP sample (measured value: 0.693). The fact that A2D-AgNP, which yielded low values in the DPPH and ABTS tests, had stronger results in this test suggests that different active groups are effective in the reduction mechanism.

Studies have shown that the antioxidant activity of AgNPs varies significantly on the basis of surface chemistry; in particular, coating agents such as phenolic and carbonyl groups increase this activity^46^. The intensity of the O–H and C = O signals observed in the FTIR spectra is thought to be related to the antioxidant activity. The electron-donating characteristics of these groups make them stand out in tests of radical scavenging and reduction. This association was validated by the KX1-AgNP sample, which produced good results in all the antioxidant assays, including the FTIR analysis. Furthermore, the morphological features of the AgNPs, including their size, shape, and aggregation, were visible in the SEM images. The homogeneous and surface-active nanoparticle structure of the KX1-AgNP sample supports its effective activity in antioxidant tests. Furthermore, the KX1-AgNP and A2B-AgNP samples demonstrated antimicrobial effects that paralleled their high DPPH activity. This may be related to the capacity of nanoparticles to target microbial cells via oxidative stress^47^.

The data revealed that the antioxidant activity of AgNPs depends not only on their chemical structure but also on their morphological properties and surface functional groups. In particular, the KX1-AgNP sample stands out as a strong candidate for potential biomedical applications, exhibiting the highest activity in all the antioxidant analyses. However, although AgNPs obtained from some microbial strains exhibit significant antioxidant properties, compared with ascorbic acid, a common antioxidant, AgNP activity has been shown to be lower.

### Cytotoxicity of AgNPs in fibroblasts

**Fig. 9:**
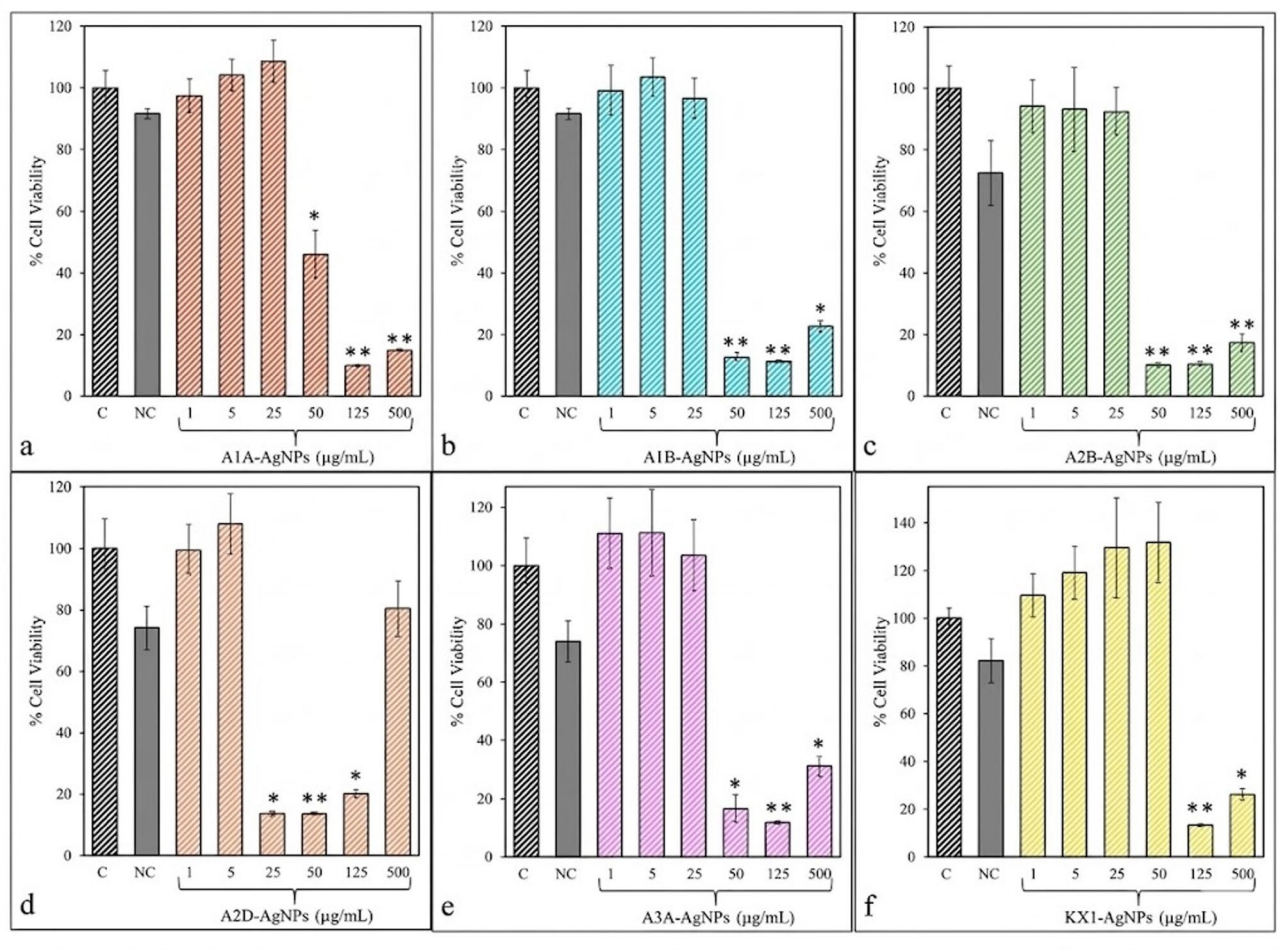
Dose-dependent effects of A1A-AgNP (**a**), A1B-AgNP (**b**), A2B-AgNP (**c**), A2D-AgNP (**d**), A3A-AgNP (**e**), KX1-AgNP (**f**), C: Control, and NC: DMSO %1 on fibroblast cell viability (%) determined by MTT assay. Data are expressed as mean ± SD (n = 3). **Statistical analysis was performed using one-way ANOVA followed by Tukey’s post hoc test (*p < 0.05, *p* < 0.01 vs. control).

An MTT cytotoxicity assay was used to assess how various AgNP concentrations affect the viability of fibroblasts. Figure 9 displays the MTT cytotoxicity graphs of the AgNPs on fibroblasts at various doses. As a result, the studied nanoparticles reduced cell viability to varying degrees on the basis of the organism that produced them. A1A-AgNPs, A1B-AgNPs, A2B-AgNPs, and A3A-AgNPs reduced cell viability at a concentration of 50 µg/mL (i < 0.05). When A2D-AgNPs or KX1-AgNPs were applied, a decrease in cell viability occurred at concentrations of 25 µg/mL and 125 µg/mL, respectively (*p* < 0.05). The viabilities of the cells treated with A1A-AgNPs at 50 µg/mL and 125 µg/mL were 46.03% and 9.99%, respectively, showing a significant dose-dependent cytotoxic effect (*p* < 0.01). A3A-AgNPs were shown to have a cell viability of 11.83% and 31.21% at doses of 125 µg/mL and 500 µg/mL, respectively, which were also statistically significant compared to the control group (*p* < 0.05). Similarly, the viability of KX1-AgNP-treated cells was 13.25% and 26.26% at the same concentrations, indicating a significant cytotoxic response (*p* < 0.05). Low doses of AgNPs may have had a stimulatory effect on fibroblast cells, and therefore cell viability may have been higher than controlled. However, these increases were not statistically significant (*p* > 0.05). These results show that the produced AgNPs have different cytotoxic effects on fibroblast cells. AgNPs exhibit tremendous variability in size, shape, and surface chemistry. Therefore, this variability can lead to changes in the biological activity and application dose of AgNPs. Furthermore, the findings suggest that AgNPs may find utility in pharmacotherapeutic settings, such as wound healing or anticancer applications^48^. In some treatments, cell viability values slightly exceeding 100% were observed. This phenomenon has also been reported in previous nanoparticle cytotoxicity studies and may be attributed to the stimulation of cellular metabolic activity at low nanoparticle concentrations. In addition, mild proliferative responses of fibroblast cells or interactions between nanoparticles and the colorimetric assay reagents may contribute to this effect. Such responses are commonly interpreted as a hormetic effect, where low concentrations of nanoparticles promote cellular activity rather than inducing cytotoxicity. Therefore, viability values slightly above 100% do not necessarily indicate experimental error but may reflect increased metabolic activity of the cells under certain exposure conditions^49–52^.

### Preparation of agar-based films and cheese packaging test

**Fig. 10:**
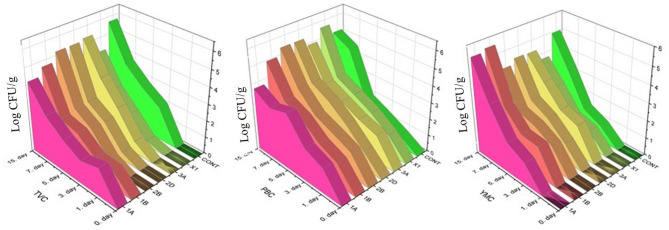
Graphs showing TVC: total viable count (lgCFU/g), YMC: yeast-mold count (lgCFU/g) and PBC: psychrotrophic bacteria count (lgCFU/g) in control cheese samples uncoated (control) and coated with 1A, 1B, 2B, 2D, 3A, or X1 films.

All the groups presented an increase in total viable count (TVC, log CFU/g) during the storage period, although the rate and degree of this increase varied depending on the treatment. Group X1 had the greatest microbial stability during storage, maintaining the lowest microbial load (4.14 log CFU/g) on days 3–5 and 15. However, group 3A had the worst microbial performance, with high TVC values that were comparable to those of the control group, particularly on days 7 and 15. When the PBC: psychrotrophic bacteria count results were analyzed, all the groups’ PBC values increased during the storage period; however, on days 1–3, 1B, X1, and the control groups presented lower values, whereas 1A and 2B presented higher psychrotroph loads. The PBC increased more quickly during the later storage days, with the 1A group showing the lowest value (3.69 log CFU/g) on day 15 and the X1 group showing the highest value (5.61 log CFU/g), suggesting that the treatments had varying effects on the growth of psychrotrophic bacteria. In the yeast-mold count (YMC) analysis, the YMC values increased in all the groups during the storage period, but the growth curves varied significantly between the treatments. Group X1 had the lowest YMC value (3.69 log CFU/g), particularly on day 15, and was the most effective treatment for limiting yeast-mold growth. In contrast, the 1A (5.34 log CFU/g) and 1B (5.50 log CFU/g) treatments had the least effect on the yeast-mold growth control, resulting in the highest YMC values. In general, when all the microbial parameters were evaluated, the X1 treatment clearly outperformed the other treatments in terms of improving microbial stability. The results are shown in Fig. 10.

In the control group, the TVC, PBC, and YMC values increased over time during storage, which is completely consistent with the microbial proliferation trends reported in the literature for cheese or similar dairy products stored at low temperatures. Silver nanoparticles (AgNPs) have become increasingly popular in active food packaging because of their broad-spectrum antimicrobial properties. In fact, packaging materials containing agar-hydrogel/AgNPs considerably decreased the load of spoilage-causing microflora in Fior di Latte cheese, especially *Pseudomonas* spp., according to Incoronato et al.,^53^. Because of their broad-spectrum activity against both gram-positive and gram-negative microorganisms, controlled silver ion release, and high surface-to-volume ratio, AgNPs have a powerful effect^19^. In the literature, it is emphasized that AgNP-enriched films significantly suppress biofilm formation, microbial growth and contamination on the food surface owing to controlled ion release, thus offering an effective strategy to extend the shelf-life of foods^19^. However, some studies have reported that AgNP-containing packaging may migrate silver into food, which may pose potential toxicological risks^54^.

## Conclusion

This study used liquid waste produced during the manufacturing of bacterial cellulose by vinegar bacteria to establish a novel and sustainable method for the green synthesis of AgNPs. The synthesized AgNPs were successfully characterized via XRD, SEM, and FTIR analyses, and their spherical morphology and crystalline structures were confirmed. Biological evaluations revealed that some nanoparticle variants, particularly KX1-AgNPs and A2B-AgNPs, exhibited antioxidant and antimicrobial activities and demonstrated concentration-dependent cytotoxic effects on cell viability. Furthermore, with the increasing prevalence of AgNP-based active packaging systems, careful consideration must be given to the potential migration of silver ions or nanoparticles into food matrices. Indeed, some studies indicate that such migration could potentially cause toxicological effects. In this context, it is important that future studies include comprehensive safety assessments to ensure that materials containing AgNP can be used safely and responsibly in applications that come into contact with food.

In addition to the structural characterization results, the DLS and zeta potential analyses further support the functional stability and applicability of the biosynthesized nanoparticles. The hydrodynamic diameters obtained from DLS measurements (85–179 nm), together with PDI values ranging from 0.274 to 0.402, indicate moderately monodisperse to heterogeneous nanoparticle populations depending on the bacterial isolate used during synthesis. These differences highlight the role of strain-specific extracellular metabolites in controlling nucleation and growth processes during nanoparticle formation. Furthermore, the negative zeta potential values (− 12.3 to − 31.8 mV) demonstrate that the nanoparticles possess moderate to high colloidal stability due to electrostatic repulsion between particles. This negative surface charge is most likely associated with the adsorption of biomolecules such as proteins, polysaccharides, and organic acids originating from the culture supernatant of acetic acid bacteria. Such naturally occurring biomolecular capping agents not only stabilize the nanoparticles but may also enhance their interaction with microbial cell membranes and reactive oxygen species, thereby contributing to their biological activities.

The biosynthesis of AgNPs in this system is likely mediated by extracellular biomolecules produced by acetic acid bacteria during bacterial cellulose fermentation. Culture supernatants of vinegar bacteria contain a variety of metabolites, including organic acids, enzymes, proteins, and polysaccharides, which may participate in nanoparticle formation. These biomolecules can act as reducing agents by donating electrons to Ag⁺ ions, leading to the formation of metallic silver nuclei. At the same time, functional groups such as hydroxyl, carbonyl, and amide groups present in these biomolecules can adsorb onto the nanoparticle surface and function as natural capping agents, preventing aggregation and enhancing colloidal stability. FTIR results indicating oxygen- and nitrogen-containing functional groups support this proposed mechanism. Moreover, these biomolecular coatings may enhance biological activity by facilitating interactions with microbial membranes and promoting the generation of reactive oxygen species, which are known to contribute to antimicrobial effects. This mechanism highlights an additional advantage of vinegar bacteria-mediated synthesis, in which microbial metabolites simultaneously function as reducing and stabilizing agents without the need for external chemical reagents.

From a green nanotechnology perspective, these findings highlight the innovative aspect of the present study. Unlike many conventional microbial or plant-based nanoparticle synthesis approaches, the current strategy utilizes the liquid residue generated during bacterial cellulose production as both the reducing and stabilizing medium. This dual functionality eliminates the need for additional chemical reagents while simultaneously valorizing a fermentation byproduct that would otherwise be discarded. Therefore, the proposed system represents a sustainable nanomaterial production platform that integrates microbial biotechnology, waste valorization, and green chemistry principles. Such an approach not only reduces environmental impact but also provides a cost-effective pathway for producing biologically active nanoparticles with potential applications in antimicrobial materials, biomedical technologies, and active food packaging systems. This research not only demonstrates the functional potential of AgNP synthesis through an environmentally friendly approach but also emphasizes the use of biological byproducts as renewable resources for nanomaterial production. Converting fermentation residues into bioactive nanoparticles represents a sustainable strategy aligned with the principles of green chemistry. To the best of our knowledge, this is one of the first studies demonstrating the use of vinegar bacteria fermentation residues as a reaction medium for the green synthesis of silver nanoparticles.

## Materials and methods

### Bacterial supply and storage

Acetic acid bacteria isolated from homemade vinegars and identified as cellulase producers were used for the production of AgNPs. The bacteria were obtained from a − 80 °C stock at the Karadeniz Advanced Technology Research and Application Centre, Ondokuz Mayıs University. The bacteria were grown on Hestrin-Schramm (HS) agar media (5 g/L yeast extract, 20 g/L glucose, 5 g/L peptone, 1.15 g/L citric acid, 2.7 g/L Na₂PO₄, 15 g/L agar) to confirm their purity^55^. The bacteria were maintained at 4 °C throughout the study.

### Screening for AgNP production

The isolates were cultivated in HS broth at 30 °C for 21 days for bacterial cellulose production. After incubation, the cellulose pellicle formed on the surface of the culture was carefully removed. The remaining culture broth was centrifuged at 10,000 × g for 10 min and subsequently filtered to obtain a cell-free supernatant. This supernatant represents the liquid waste generated during bacterial cellulose production and was used as the reaction medium for silver nanoparticle synthesis. For AgNP production, the culture supernatant was mixed with AgNO_3_ solution to obtain a final 3 mM (Milimolar) AgNO_3_ concentration. The reaction mixture was incubated at 50 °C in the dark for 48 h. The formation of silver nanoparticles was confirmed by monitoring the surface plasmon resonance using UV–Vis spectrophotometry.

### Identification of isolates on the basis of the 16S rRNA gene

DNA was isolated for the characterization of acetic acid bacteria. A Genomic DNA Purification GeneJET Kit was used for DNA isolation. The 16S ribosomal DNA sections were subjected to polymerase chain reaction (PCR) to amplify the extracted DNA with 27 forward (5'-AGAGTTTGATCMTGGCTCAG-3') and 1492 reverse (5'-TACGGYTACCTTGTTACGACTT-3') primers in accordance with the kit procedure. The reactions were performed using Thermo Scientific DreamTaq DNA Polymerase. Four microliters of genomic DNA, five microliters of 10 × buffer mixture, one microliter of deoxynucleotide triphosphate, three microliters of each primer mixture, 0.25 µl of DreamTaq (Thermo Scientific), and 33.75 µl of nuclease-free water were included in the PCR mixture. Following three minutes of initial denaturation at 95 °C, the reactions were run 40 times at 95 °C for 30 s, 55 °C for 30 s, and 72 °C for 1 min, followed by a final extension at 72 °C for 15 min. The protocol was performed with a Genepro thermal cycler (China, Bioer Technology) model TC-E-96G.

Agarose gel electrophoresis was used to evaluate the size and integrity of the PCR products at 90 V for 90 min. On a 1% agarose gel with 10 µL/mL EtBr, the DNA bands were visualized via the Chemidoc MP imaging system (Bio-Rad Laboratories, Hercules, CA). PCR products, whose densities and purities (A260/A280) were determined spectrophotometrically, were purified with the SeqLine CleanSEQ Kit and then sequenced bidirectionally via an ABI 3130 genetic analyzer (Applied Biosystems). The quality of the DNA sequences obtained with forward and reverse primers for each gene was checked via the Chromas program (Technelysium Pty. Ltd., Tewantin, Queensland, Australia) and assembled via the BioEdit program. The sequences from the EzBioCloud 16S database (https://www.ezbiocloud.net/) were subsequently compared to the 16S rRNA gene sequences of the isolates. Finally, the MEGA 6 program was used to create phylogenetic trees of the isolates via the neighbor‒joining method^56^.

### Production and recovery of AgNPs

First, AgNO_3_ was added to the supernatant separated by centrifugation (10 000 × *g*, 10 min) from a 21-day bacterial culture grown in HS broth at 30 °C and 150 rpm shaking conditions to achieve a final concentration of 3 mM. The reaction mixture was incubated at 50 °C for 48 h in the dark, then centrifuged (13.000–15.000 × g, 30 min) to precipitate the synthesized AgNPs. Furthermore, AgNPs were washed with ultra-pure water and dried in a moisture-free oven at 50 °C, and finally stored in the dark until use.

### Physicochemical characterization of AgNPs

#### Imaging with a scanning electron microscope (SEM) and EDS mapping: microstructural characterization

A JEOL JSM-7001F field emission scanning electron microscope (FESEM) running at 15 kV was used to analyze the samples after a suitable quantity of powdered AgNPs was placed on a 0.5 cm^2^ glass slide. Using an X-Max 80 mm^2^ detector (Oxford Instruments, UK) built into the FESEM, EDS mapping was carried out on the same slide^57^.

#### Attenuated total reflection-fourier transform infrared spectroscopy (ATR-FTIR)

The ATR-FTIR analysis of the dried AgNPs was carried out using a PerkinElmer Spectrum 100 FT-IR spectrometer (Germany) in the range of 400–4000 cm^−1^ with a resolution of 4 cm^−1^. Prior to analysis, the synthesized AgNPs were centrifuged and washed several times with distilled water to remove unbound biomolecules and residual impurities, followed by drying at room temperature. A small amount of dried nanoparticle powder was directly placed onto the ATR crystal surface without further preparation. Spectra were recorded by averaging multiple scans to improve the signal-to-noise ratio^58^.

The obtained spectra were used to identify functional groups associated with biomolecules responsible for the reduction and stabilization of AgNPs. In particular, characteristic absorption bands corresponding to O–H, C = O, C–O, and N–H functional groups were analyzed to evaluate the involvement of proteins, polysaccharides, or other organic compounds in nanoparticle synthesis and capping. ATR-FTIR is widely used for nanoparticle surface characterization due to its minimal sample preparation and ability to probe molecular interactions at the surface level.

#### X-ray diffraction (XRD) analysis

The crystalline structure of the synthesized AgNPs was determined by X-ray diffraction (XRD) analysis using a Rigaku SmartLab diffractometer (Rigaku SmartLab Beijing Co., China) equipped with Cu Kα radiation (λ = 1.5406 Å) and a Cu Kβ filter. The powdered AgNP samples were prepared by drying and finely grinding prior to analysis to ensure homogeneity. The diffraction patterns were recorded over a 2θ range of 10–80° at a scanning rate of 2°/min under operating conditions of 40 kV and 30 mA in continuous scanning mode.

The obtained diffraction peaks were indexed and compared with standard Joint Committee on Powder Diffraction Standards (JCPDS) data to confirm the face-centered cubic (FCC) crystalline structure of silver^59^. The characteristic diffraction peaks corresponding to the (111), (200), (220), and (311) planes were used to verify the formation of crystalline AgNPs. Additionally, the average crystallite size was estimated using the Debye–Scherrer equation based on the full width at half maximum (FWHM) of the most intense diffraction peak. XRD analysis provides essential information about crystallinity, phase purity, and structural properties of nanoparticles.

#### Dynamic light scattering (DLS) and zeta potential (ζ)

Size distribution, mean diameter, and zeta-potential (ζ) of AgNPs in ultra-pure water (refractive index: 1.330, viscosity: 0.8872) were analyzed using Zetasizer Nano ZS (Malvern Panalytical, UK) at 25 °C. A 2 mL AgNPs suspension (1 mg/mL) in a quartz cuvette was laser-illuminated, and scattered light fluctuations were analyzed to determine particle size via Brownian motion.

### Antimicrobial activity analyses

Several of the tested microorganisms, including *Bacillus cereus*, *Escherichia coli*, *Yersinia enterocolitica*, *Klebsiella pneumoniae*, and *Pseudomonas aeruginosa*, are commonly associated with food contamination, spoilage, or foodborne infections. Therefore, these microorganisms were selected to evaluate the potential applicability of the synthesized AgNPs in food preservation and active packaging systems. Gram-positive bacteria, including *S. aureus* (ATCC 43300—methicillin-resistant), *S. aureus* (ATCC 25923–methicillin-sensitive), *B. cereus* (NTCC 7464), *B. subtilis* (ATCC 6633), and *E. faecalis* (ATCC 29212), were used. Gram-negative bacteria, including *E. coli* (ATCC 13462—ESBL-positive), *E. coli* (ATCC 25922—ESBL-negative), *K. pneumoniae* (ATCC 700603), *P. aeruginosa* (ATCC 9027), *Y. enterocolitica* (ATC 23717), *A. baumannii* (ATCC 19606), and *C. albicans* yeast, were used. For the experiments, fresh microorganism cultures were cultivated in nutrient broth media at 35 °C while being shaken at 150 rpm. Streptomycin (10 µg) served as the positive control, whereas buffered peptone water used as the solvent for the nanoparticles was employed as the negative control in the antimicrobial activity assay.

#### Agar well diffusion method

100-µL aliquots of bacterial and yeast suspensions were distributed onto Mueller–Hinton agar plates after being adjusted to 10⁸ colony-forming units (CFU/ml). Wells 5 mm in diameter were cut into Mueller Hinton agar (MHA) medium and filled with 50 µL of silver nanoparticle solution (100 μg/ml). The mixture was then preincubated for 10 min at room temperature in an ultrasonic bath. The widths of the inhibition zones that developed around the wells were measured following a 24-h incubation period at 37 °C. Antibiotic discs containing 10 µg of streptomycin served as controls^60^.

#### Minimum inhibition concentration (MIC)

The microdilution method was applied. Briefly, nutrient broth media containing nanoparticles diluted to final concentrations of 16–500 µg/mL were inoculated with approximately 10^5^ microorganisms (5 µL) and incubated at 35 °C for 24 h. Changes in turbidity in the culture were subsequently measured on the basis of microbial growth. The lowest concentration of AgNPs that did not allow microbial growth was defined as the MIC. The experiments were conducted in triplicate on sterile 96-well plates^61^.

#### Minimum lethal concentration (MLC)

To determine the minimum lethal concentration (MLC), 5 µL aliquots from MIC wells showing no visible growth were plated onto Mueller–Hinton agar (MHA). Following a 24-h incubation period at 37 °C. The MLC was defined as the lowest concentration of AgNPs that resulted in no microbial growth on the agar plates, indicating bactericidal activity. All MIC and MLC values were expressed as µg/mL^60^.

### Antioxidant activity analyses

#### DPPH free radical scavenging

The methods described by Konen-Adigüzel et al. (2018) and Brand-Williams et al. (1995) were used to determine the DPPH radical scavenging activity^62,63^. This technique is based on antioxidants reducing the DPPH radical, which decreases the purple hue. The 0.04% DPPH solution was combined with AgNPs made in ethanol at various concentrations (50–800 µg/mL), and the mixture was left to sit at room temperature in the dark for half an hour. As a positive control, ascorbic acid was used, while a blank sample (without AgNP) served as the negative control. Absorbance was measured at 517 nm. The following formula was used to determine the percentage of free radical scavenged (% inhibition):

$$\% {\text{ Inhibition}} = \left( {{\mathrm{A}}_{{{\mathrm{contro}}}} {\mathrm{l}}{-}{\mathrm{A}}_{{{\mathrm{sample}}}} } \right)/{\mathrm{A}}_{{{\mathrm{control}}}} \times { 1}00$$

(A_control_: Absorbance of the control; A_sample_: Absorbance of the sample).

#### ABTS^·+^ radical scavenging

With few changes, the method of Re et al. (1999) was used to assess the ABTS⁺ radical scavenging activity^48,64^. The ABTS^.^⁺ radical solution obtained by the reaction of 7 mM ABTS and 2.45 mM potassium persulfate was incubated in the dark at room temperature for 12–16 h. To obtain an absorbance value of 0.70 ± 0.02 at 734 nm, the produced ABTS solution was diluted with methanol prior to examination. AgNPs made in ethanol at various concentrations were combined with 1 ml of ABTS solution, and the mixture was then incubated for half an hour. The absorbances at 734 nm were measured for all the samples. Ascorbic acid was used as a positive control. The percentage inhibition of ABTS radical scavenging activity was calculated via the following formula:

#### Fe^+3^—Fe^+2^ reduction capacity

To determine the iron-reducing capacity of the extracts, the techniques of Oyaizu and Kaygisiz et al. were employed^65,66^. Different quantities of AgNPs made in ethanol were introduced into test tubes, and the contents were adjusted with distilled water to a total volume of 1 mL. Next, 2.5 mL of 1% potassium ferricyanide [K_3_Fe(CN)_6_] and 2.5 mL of phosphate buffer (0.2 M, pH 6.6) were added to the tubes. A total of 2.5 mL of TCA (10%) solution was added to the reaction mixture after it had been incubated for 20 min at 50 °C. The top phase of the solution (2.5 mL) was extracted. The absorbance values were then measured at 700 nm after 0.5 mL of FeCl_3_ (0.1%) and 2.5 mL of distilled water were added.

### Cytotoxicity of AgNPs in fibroblasts

Dulbecco’s modified Eagle’s medium (DMEM), which was supplemented with 10% fetal bovine serum, 100 U/mL penicillin, and 100 µg/mL streptomycin, was used to cultivate the fibroblasts. The cells were cultured in 5% CO₂ at 37 °C until 80–90% confluence was achieved. Throughout the incubation period, the culture medium was replaced every two to three days.

After adequate growth was attained, 100 µL of cell suspension containing approximately 3 × 10^4^ cells/mL was added to the wells of the 96-well microplates. The plates were then incubated for 24 h at 22 °C in 5% CO₂ to promote adhesion. Following incubation, AgNPs were added to the plates in the same volume as the 2X medium for exposure. Cytotoxicity was assessed via the MTT assay after exposure^62,67^.

### Preparation of agar-based films and cheese packaging test

**Fig. 11:**
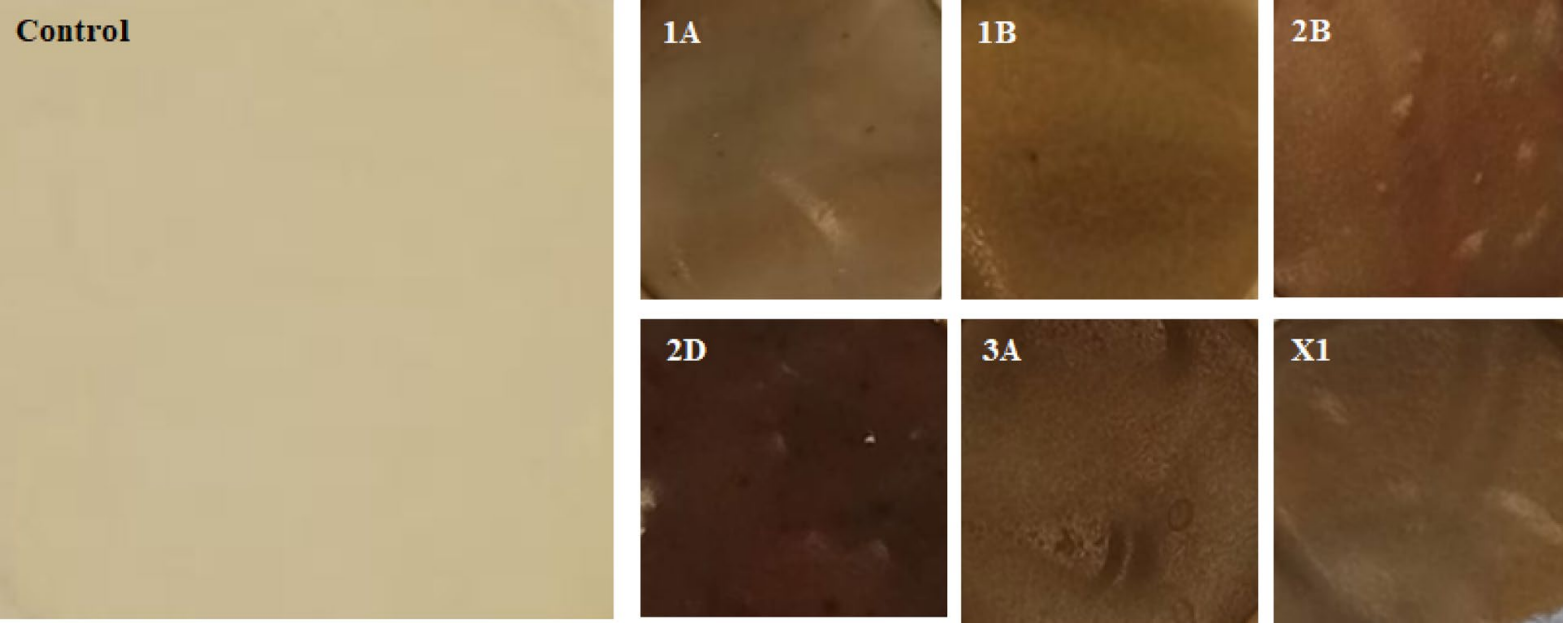
Images of agar-based films prepared with and without the addition of AgNPs 1A, 1B, 2B, 2D, 3A, and X1.

Agar films were fabricated via the solution-casting technique. Briefly, 2 g of agar was dissolved in 200 mL of distilled water, and 1.2 g of glycerol (corresponding to 30 wt% of the biopolymer) was added as a plasticizer. The mixture was continuously stirred at 90 °C for 1 h until complete homogenization. Subsequently, silver nanoparticles (0 and 1 wt%) were incorporated into the solution under vigorous mixing. The final mixture was cast onto clean glass plates and dried in an oven at 55 ± 5 °C for 24 h to obtain uniform films^68^. The formed AgNp films are shown in Fig. 11.

Cheese samples of approximately 1 g were prepared and completely wrapped with agar-based films that had been sterilized by UV exposure (30 min per side). Each sample was placed in sterile polyethylene zip-lock bags and stored aerobically at + 4 °C for 15 days. Control samples without film wrapping were prepared under the same conditions. Microbial spoilage during storage was evaluated on days 0, 7, and 15 by determining the total viable count (TVC), psychrotrophic bacteria count (PBC), and yeast and mold count (YMC)^69^.

#### Data analysis

All statistical analyses were performed using SPSS, version 20, One-way analysis of variance (ANOVA) was applied to determine significant differences between experimental groups and the control group. When significant differences were detected, Duncan’s multiple range test was used to determine changes between groups. Correlation analyses were performed to evaluate the relationships between parameters. If the *p* value obtained from all the statistical analyses was < 0.05, the difference was considered significant. The software program OriginPro 2023 was used to create the graphs (OriginLab Corporation, USA).

## Supplementary Information

Below is the link to the electronic supplementary material.

Supplementary Material 1

Supplementary Material 2

Supplementary Material 3

Supplementary Material 4

Supplementary Material 5

## Data Availability

The datasets used and/or analysed during the current study available from the corresponding author on reasonable request. EDS, SEM, FTIR and XRD data generated or analysed during this study are included in this published article and its supplementary information files. In addition, 16S rRNA partial sequences of isolates 1A, 1B, 2B, 2D, 3A, and X1 are deposited in the GenBank database of NCBI with accession numbers PV341489 (https://www.ncbi.nlm.nih.gov/nuccore/PV341489.1/), PV341624 (https://www.ncbi.nlm.nih.gov/nuccore/PV341624), PV341496 (https://www.ncbi.nlm.nih.gov/nuccore/PV341496), PV341505 (https://www.ncbi.nlm.nih.gov/nuccore/PV341505), PV341506 (https://www.ncbi.nlm.nih.gov/nuccore/PV341506), and PV341662 (https://www.ncbi.nlm.nih.gov/nuccore/PV341662), respectively.
