# Supplementary material for: Sustainable biosynthesis of silver nanoparticles from vinegar bacteria fermentation waste: characterization, bioactivity and food packaging potential
Source: Sci Rep. 2026 May 14;16:22000. doi: 10.1038/s41598-026-53384-9 (PMC13365466; doi:10.1038/s41598-026-53384-9)
Supplement: Supplementary file 3 — Supplementary Material 3 [file 41598_2026_53384_MOESM3_ESM.zip › Edsreports/Project 1_1A_2024-12-09_13-52-04.docx]

Project Notes

Click here to enter text.

Specimen Notes

Click here to enter text.


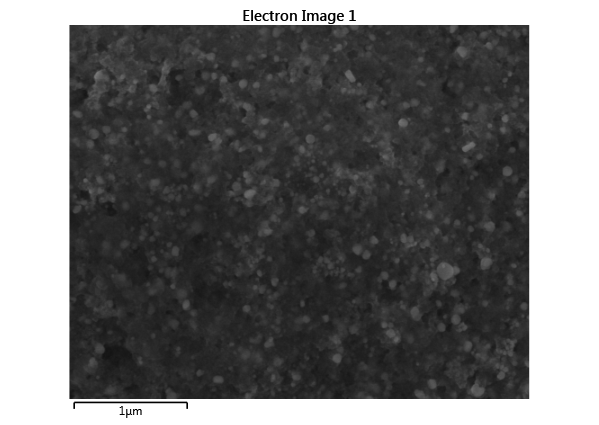


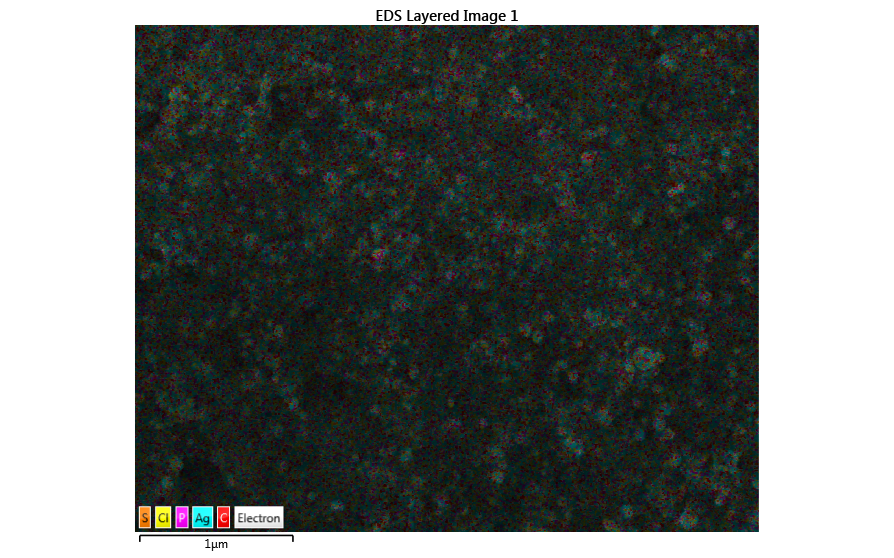


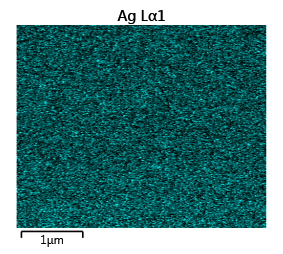

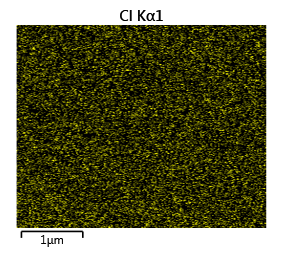

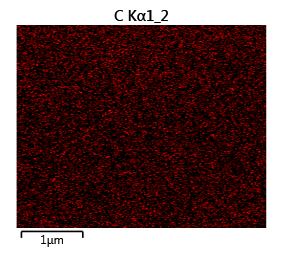

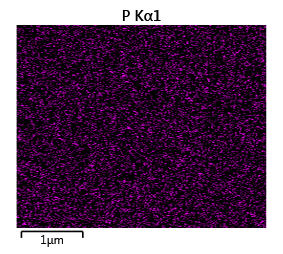

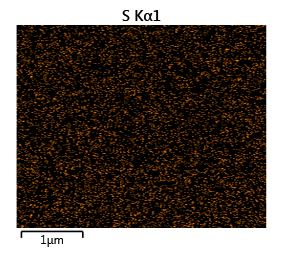

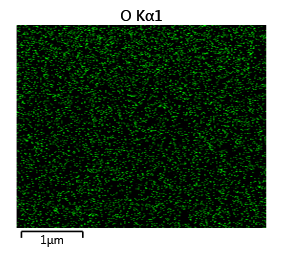

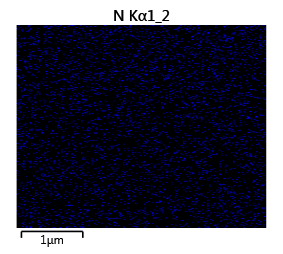


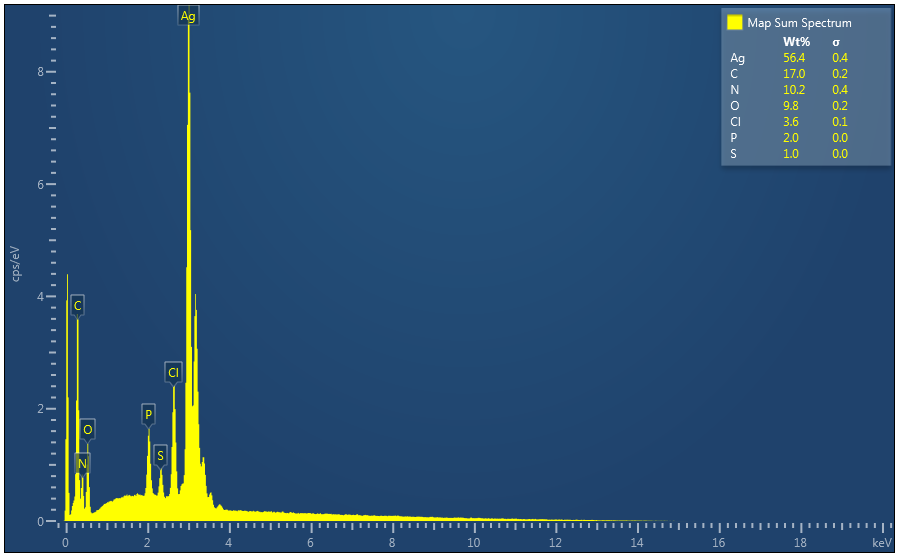


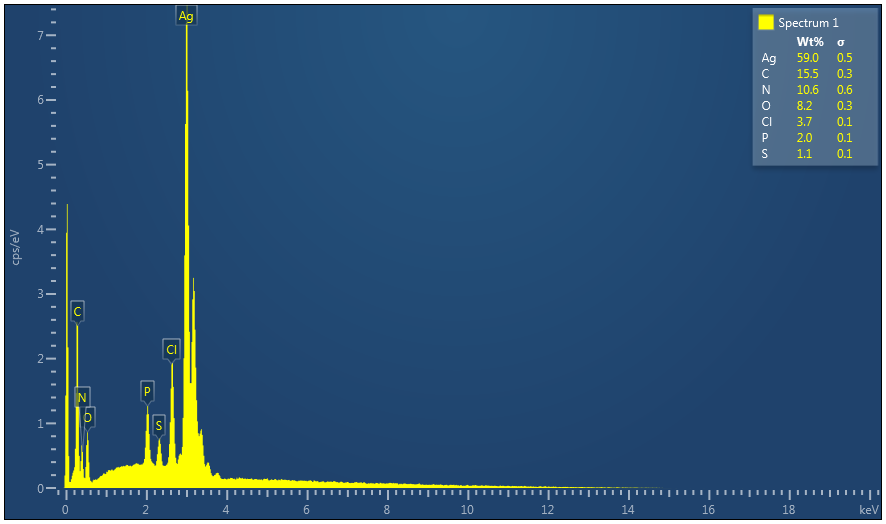


| Element | Line Type | Apparent Concentration | k Ratio | Wt% | Wt% Sigma | Standard Label | Factory Standard | Standard Calibration Date |
| --- | --- | --- | --- | --- | --- | --- | --- | --- |
| C | K series | 0.96 | 0.00959 | 15.48 | 0.29 | C Vit | Yes |  |
| N | K series | 1.21 | 0.00215 | 10.58 | 0.59 | BN | Yes |  |
| O | K series | 0.49 | 0.00163 | 8.17 | 0.29 | SiO2 | Yes |  |
| P | K series | 0.43 | 0.00240 | 2.01 | 0.07 | GaP | Yes |  |
| S | K series | 0.16 | 0.00142 | 1.08 | 0.06 | FeS2 | Yes |  |
| Cl | K series | 0.56 | 0.00491 | 3.67 | 0.09 | NaCl | Yes |  |
| Ag | L series | 7.17 | 0.07172 | 59.01 | 0.50 | Ag | Yes |  |
| Total: |  |  |  | 100.00 |  |  |  |  |
