# Supplementary material for: Sustainable biosynthesis of silver nanoparticles from vinegar bacteria fermentation waste: characterization, bioactivity and food packaging potential
Source: Sci Rep. 2026 May 14;16:22000. doi: 10.1038/s41598-026-53384-9 (PMC13365466; doi:10.1038/s41598-026-53384-9)
Supplement: Supplementary file 3 — Supplementary Material 3 [file 41598_2026_53384_MOESM3_ESM.zip › Edsreports/Project 1_1B_2024-12-09_13-51-45.docx]

Project Notes

Click here to enter text.

Specimen Notes

Click here to enter text.


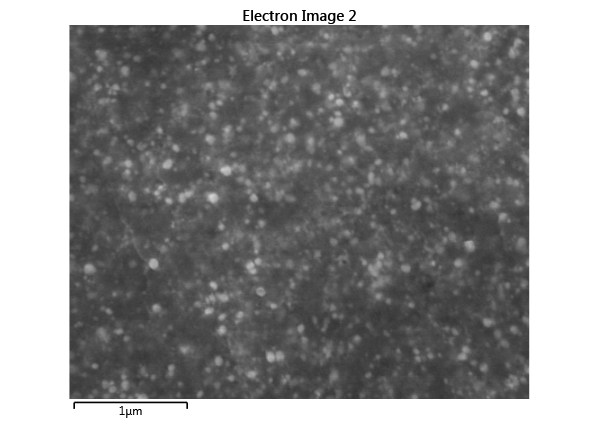


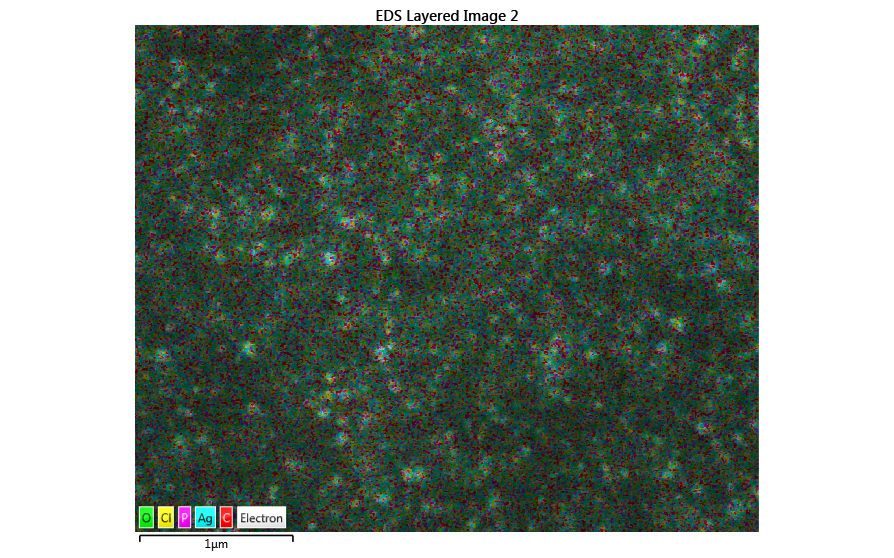


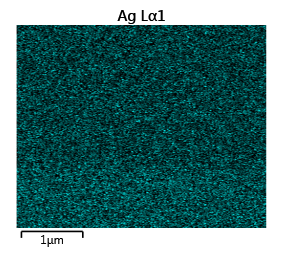

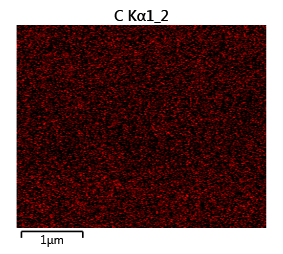

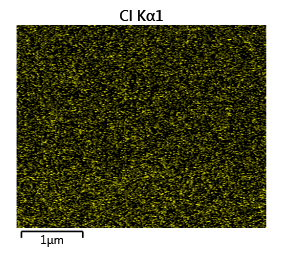

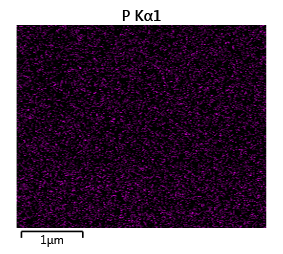

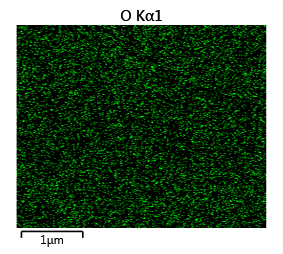

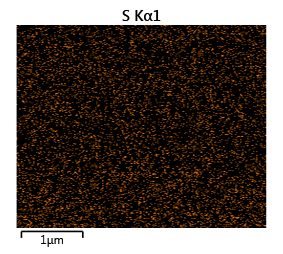

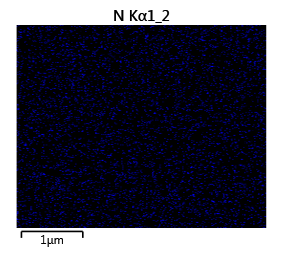


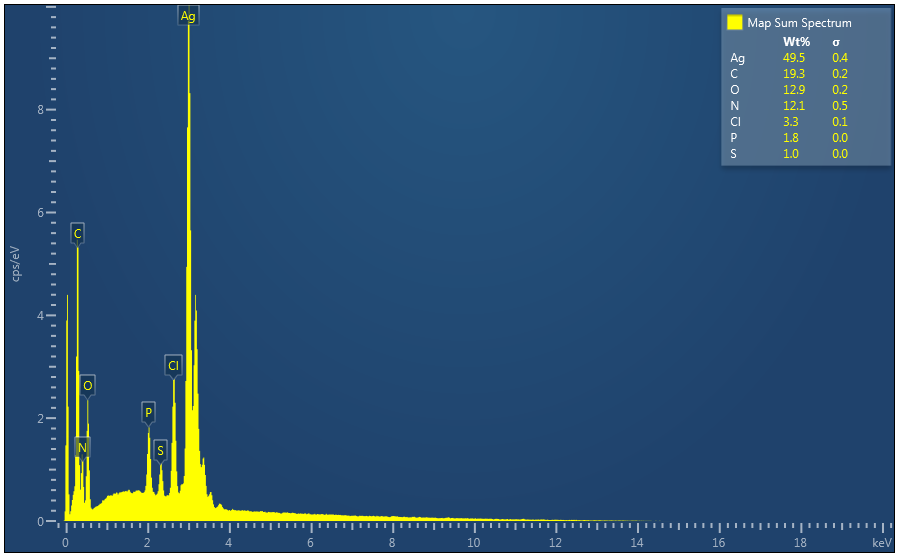


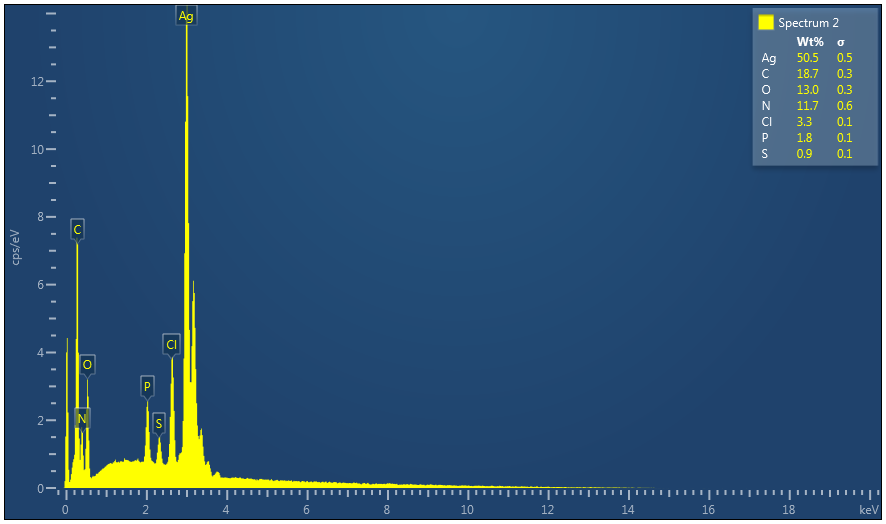


| Element | Line Type | Apparent Concentration | k Ratio | Wt% | Wt% Sigma | Standard Label | Factory Standard | Standard Calibration Date |
| --- | --- | --- | --- | --- | --- | --- | --- | --- |
| C | K series | 2.71 | 0.02711 | 18.69 | 0.31 | C Vit | Yes |  |
| N | K series | 3.12 | 0.00555 | 11.70 | 0.62 | BN | Yes |  |
| O | K series | 1.84 | 0.00620 | 13.03 | 0.31 | SiO2 | Yes |  |
| P | K series | 0.89 | 0.00499 | 1.84 | 0.06 | GaP | Yes |  |
| S | K series | 0.30 | 0.00261 | 0.88 | 0.05 | FeS2 | Yes |  |
| Cl | K series | 1.15 | 0.01003 | 3.34 | 0.08 | NaCl | Yes |  |
| Ag | L series | 13.79 | 0.13787 | 50.52 | 0.47 | Ag | Yes |  |
| Total: |  |  |  | 100.00 |  |  |  |  |
