# Supplementary material for: Sustainable biosynthesis of silver nanoparticles from vinegar bacteria fermentation waste: characterization, bioactivity and food packaging potential
Source: Sci Rep. 2026 May 14;16:22000. doi: 10.1038/s41598-026-53384-9 (PMC13365466; doi:10.1038/s41598-026-53384-9)
Supplement: Supplementary file 3 — Supplementary Material 3 [file 41598_2026_53384_MOESM3_ESM.zip › Edsreports/Project 1_2B_2024-12-09_13-53-41.docx]

Project Notes

Click here to enter text.

Specimen Notes

Click here to enter text.


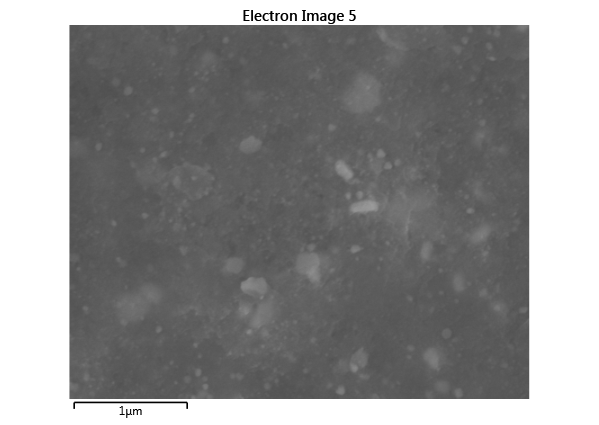


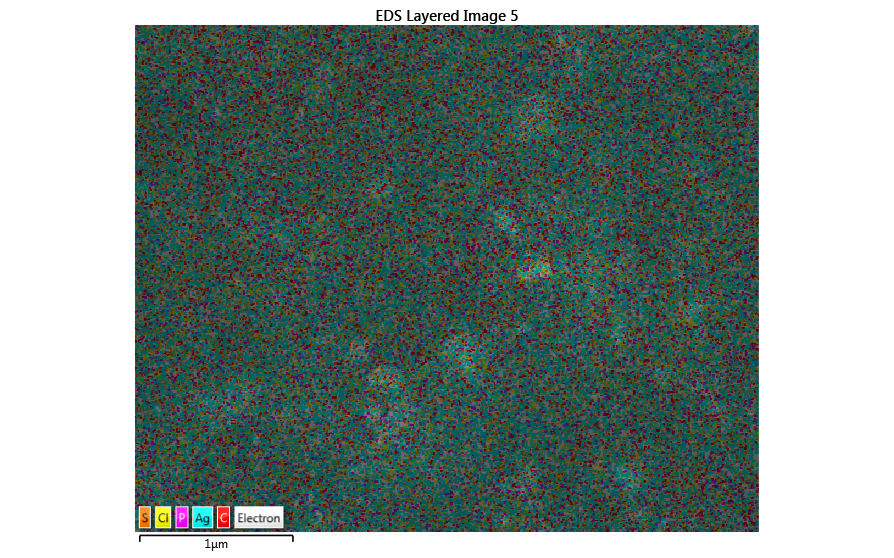


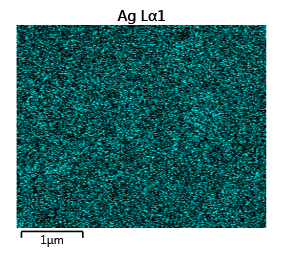

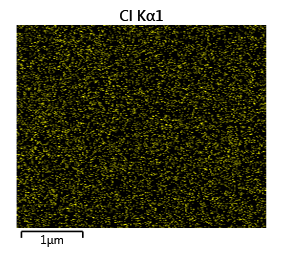

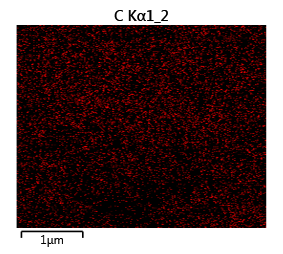

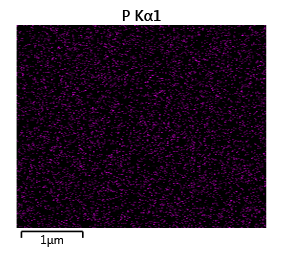

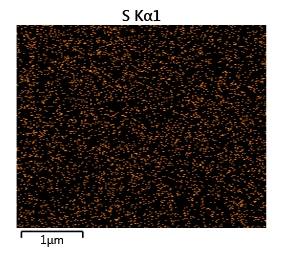

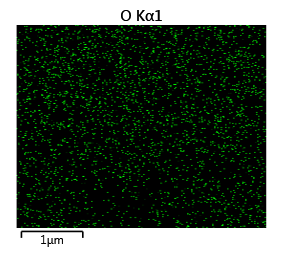

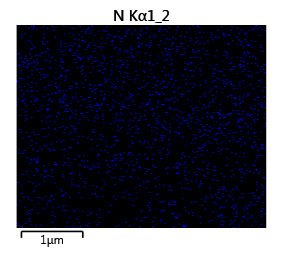


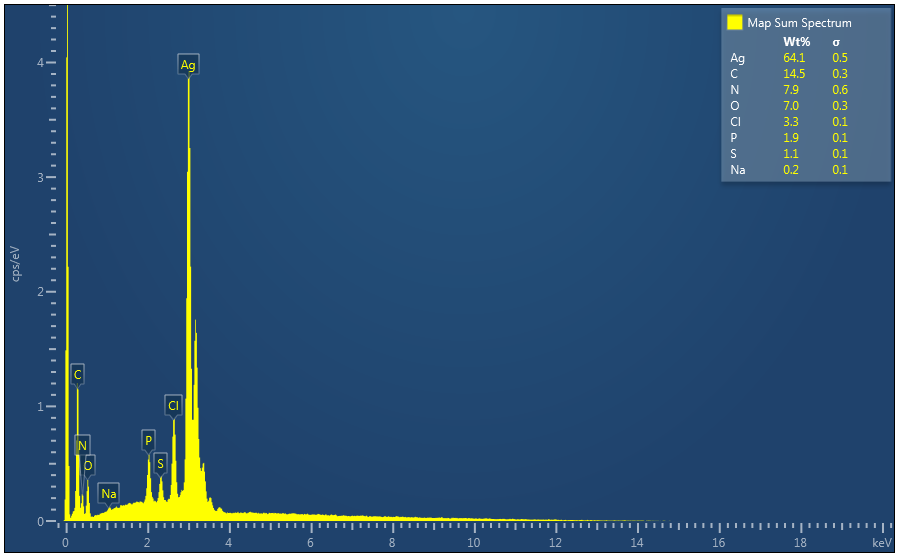


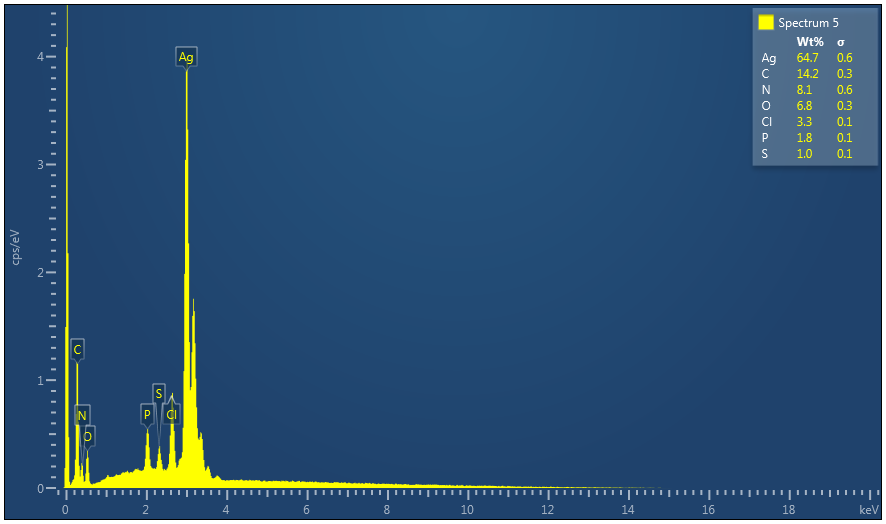


| Element | Line Type | Apparent Concentration | k Ratio | Wt% | Wt% Sigma | Standard Label | Factory Standard | Standard Calibration Date |
| --- | --- | --- | --- | --- | --- | --- | --- | --- |
| C | K series | 0.45 | 0.00449 | 14.21 | 0.31 | C Vit | Yes |  |
| N | K series | 0.43 | 0.00077 | 8.09 | 0.64 | BN | Yes |  |
| O | K series | 0.19 | 0.00065 | 6.78 | 0.30 | SiO2 | Yes |  |
| P | K series | 0.19 | 0.00106 | 1.85 | 0.08 | GaP | Yes |  |
| S | K series | 0.08 | 0.00067 | 1.05 | 0.07 | FeS2 | Yes |  |
| Cl | K series | 0.25 | 0.00215 | 3.31 | 0.10 | NaCl | Yes |  |
| Ag | L series | 3.83 | 0.03829 | 64.71 | 0.58 | Ag | Yes |  |
| Total: |  |  |  | 100.00 |  |  |  |  |
