# Supplementary material for: Sustainable biosynthesis of silver nanoparticles from vinegar bacteria fermentation waste: characterization, bioactivity and food packaging potential
Source: Sci Rep. 2026 May 14;16:22000. doi: 10.1038/s41598-026-53384-9 (PMC13365466; doi:10.1038/s41598-026-53384-9)
Supplement: Supplementary file 3 — Supplementary Material 3 [file 41598_2026_53384_MOESM3_ESM.zip › Edsreports/Project 1_2D_2024-12-09_13-53-54.docx]

Project Notes

Click here to enter text.

Specimen Notes

Click here to enter text.


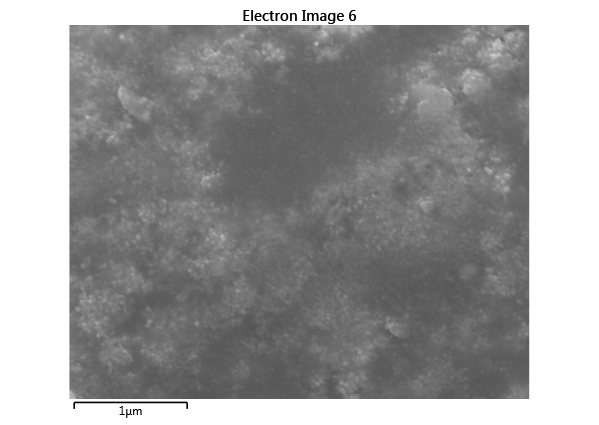


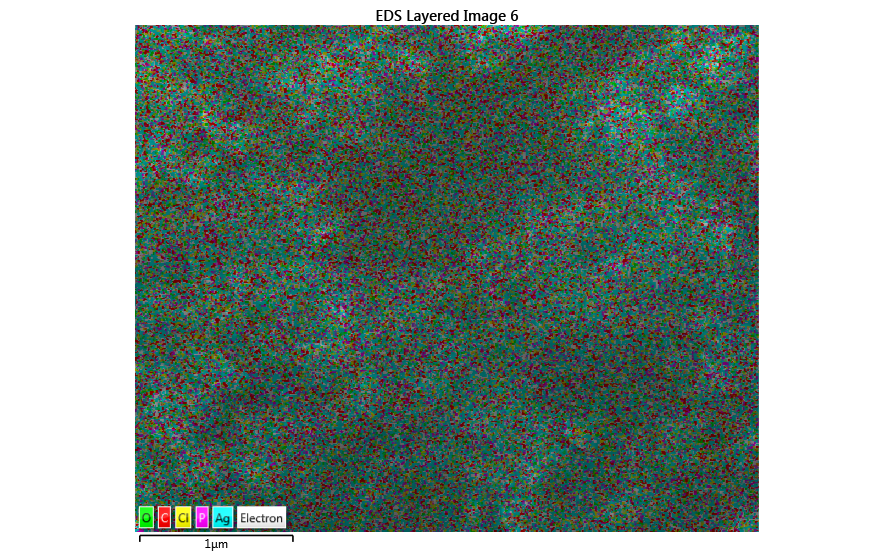


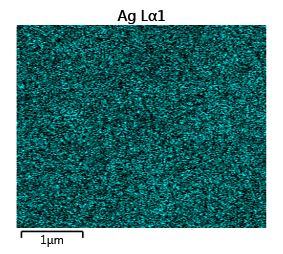

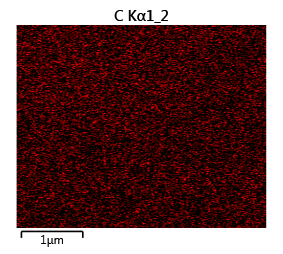

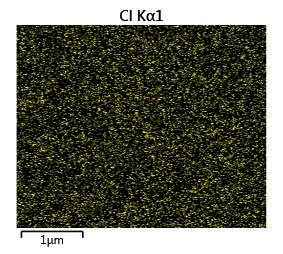

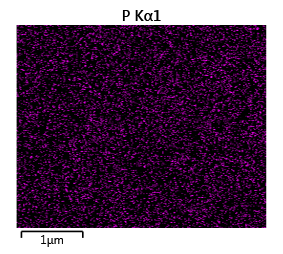

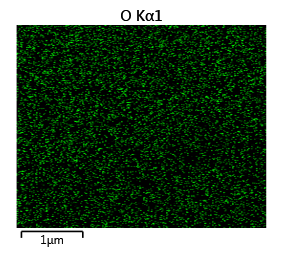

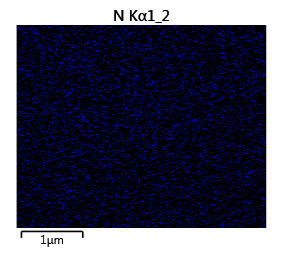

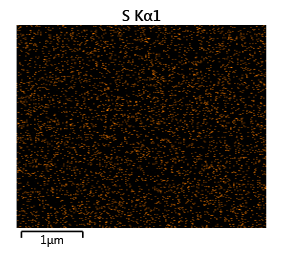


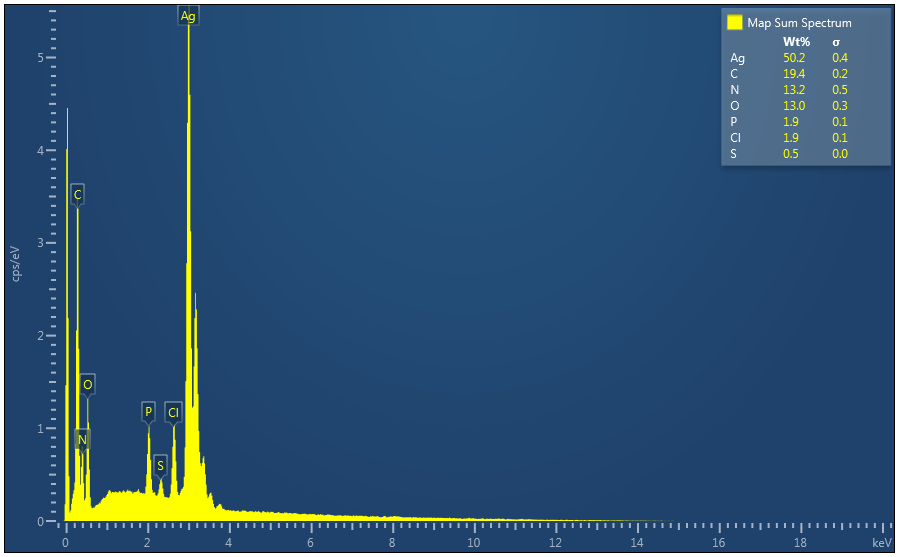


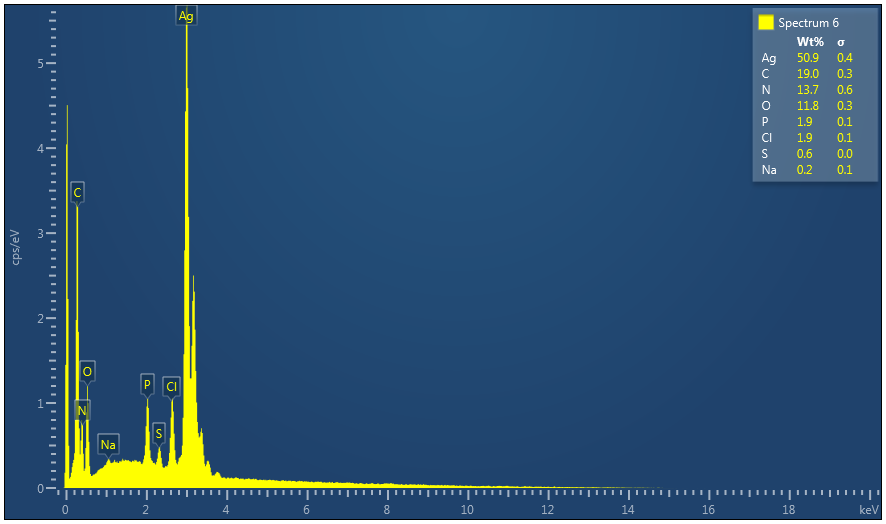


| Element | Line Type | Apparent Concentration | k Ratio | Wt% | Wt% Sigma | Standard Label | Factory Standard | Standard Calibration Date |
| --- | --- | --- | --- | --- | --- | --- | --- | --- |
| C | K series | 1.24 | 0.01245 | 18.99 | 0.27 | C Vit | Yes |  |
| N | K series | 1.48 | 0.00263 | 13.72 | 0.55 | BN | Yes |  |
| O | K series | 0.64 | 0.00217 | 11.76 | 0.29 | SiO2 | Yes |  |
| Na | K series | 0.02 | 0.00010 | 0.20 | 0.06 | Albite | Yes |  |
| P | K series | 0.37 | 0.00205 | 1.92 | 0.06 | GaP | Yes |  |
| S | K series | 0.09 | 0.00074 | 0.63 | 0.04 | FeS2 | Yes |  |
| Cl | K series | 0.25 | 0.00220 | 1.85 | 0.06 | NaCl | Yes |  |
| Ag | L series | 5.53 | 0.05526 | 50.93 | 0.43 | Ag | Yes |  |
| Total: |  |  |  | 100.00 |  |  |  |  |
