# Supplementary material for: Sustainable biosynthesis of silver nanoparticles from vinegar bacteria fermentation waste: characterization, bioactivity and food packaging potential
Source: Sci Rep. 2026 May 14;16:22000. doi: 10.1038/s41598-026-53384-9 (PMC13365466; doi:10.1038/s41598-026-53384-9)
Supplement: Supplementary file 3 — Supplementary Material 3 [file 41598_2026_53384_MOESM3_ESM.zip › Edsreports/Project 1_3A_2024-12-09_13-54-07.docx]

Project Notes

Click here to enter text.

Specimen Notes

Click here to enter text.


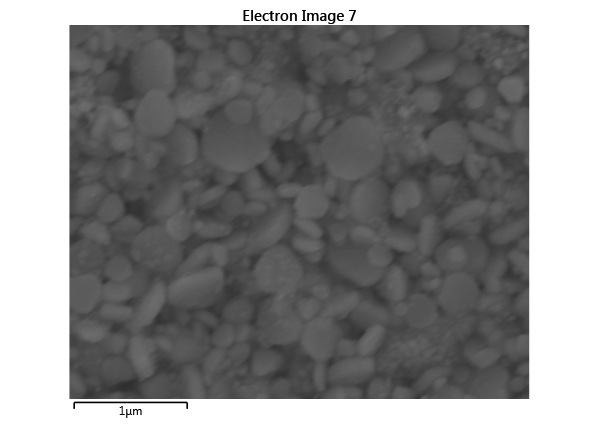


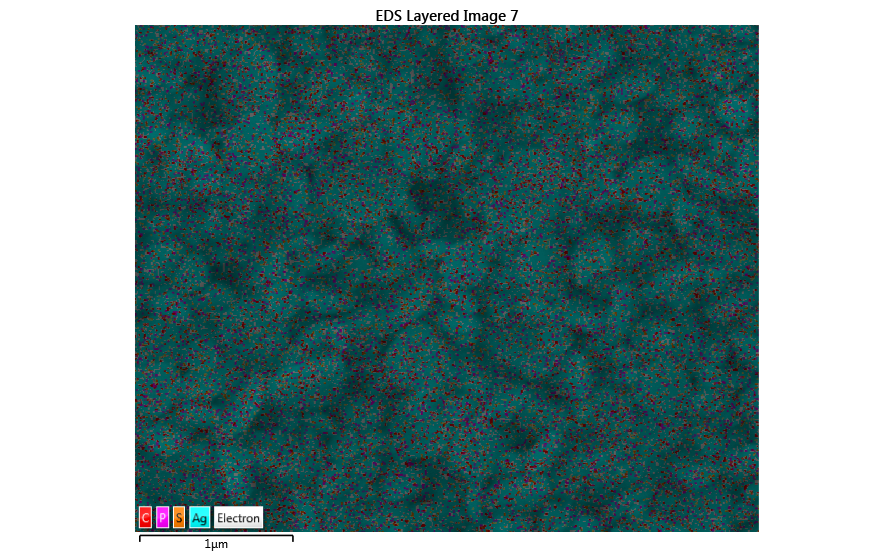


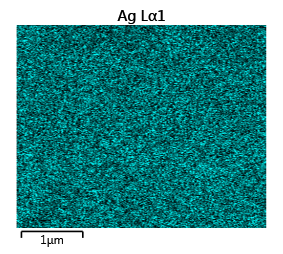

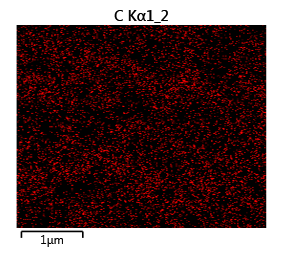

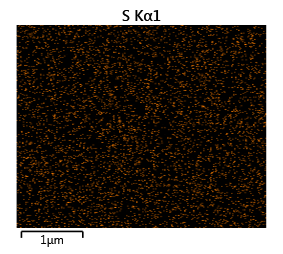

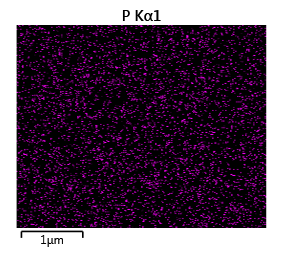


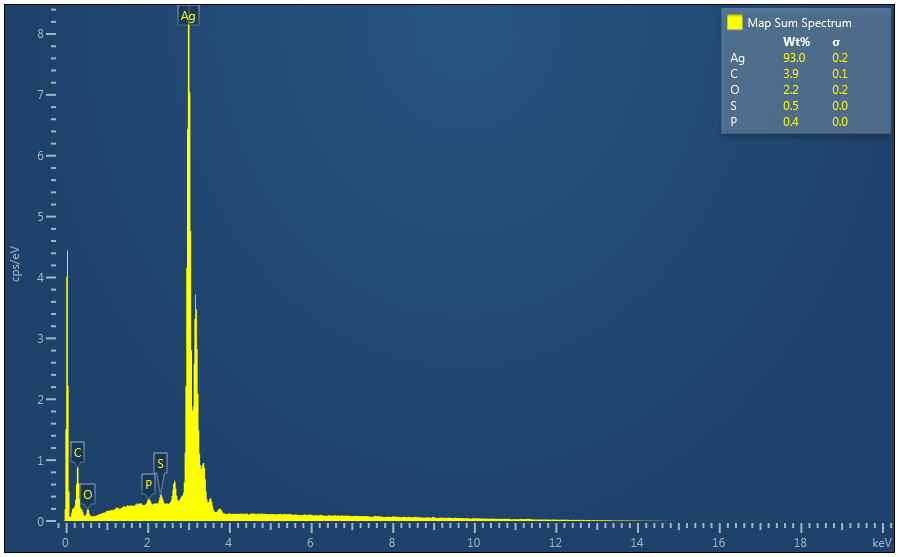


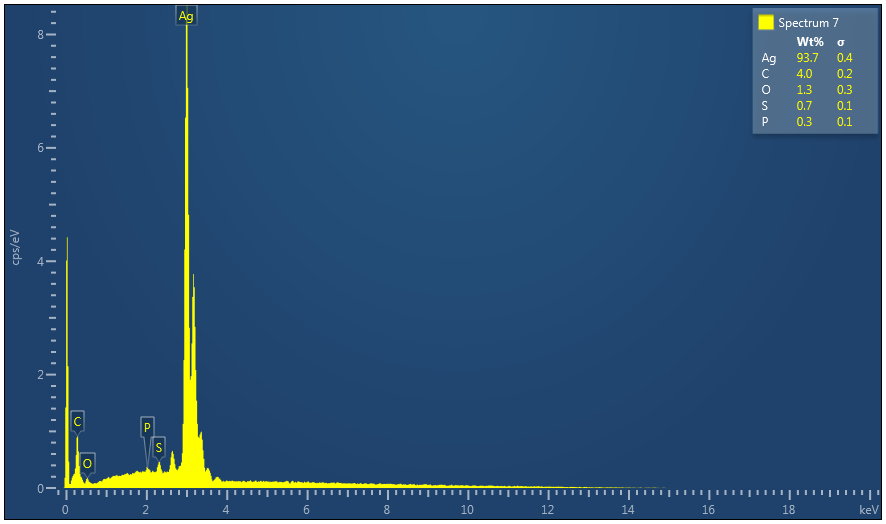


| Element | Line Type | Apparent Concentration | k Ratio | Wt% | Wt% Sigma | Standard Label | Factory Standard | Standard Calibration Date |
| --- | --- | --- | --- | --- | --- | --- | --- | --- |
| C | K series | 0.25 | 0.00251 | 3.98 | 0.23 | C Vit | Yes |  |
| O | K series | 0.05 | 0.00017 | 1.35 | 0.31 | SiO2 | Yes |  |
| P | K series | 0.04 | 0.00021 | 0.26 | 0.08 | GaP | Yes |  |
| S | K series | 0.07 | 0.00062 | 0.68 | 0.08 | FeS2 | Yes |  |
| Ag | L series | 8.31 | 0.08309 | 93.74 | 0.38 | Ag | Yes |  |
| Total: |  |  |  | 100.00 |  |  |  |  |
