# Supplementary material for: Sustainable biosynthesis of silver nanoparticles from vinegar bacteria fermentation waste: characterization, bioactivity and food packaging potential
Source: Sci Rep. 2026 May 14;16:22000. doi: 10.1038/s41598-026-53384-9 (PMC13365466; doi:10.1038/s41598-026-53384-9)
Supplement: Supplementary file 3 — Supplementary Material 3 [file 41598_2026_53384_MOESM3_ESM.zip › Edsreports/Project 1_X1_2024-12-09_13-54-18.docx]

Project Notes

Click here to enter text.

Specimen Notes

Click here to enter text.


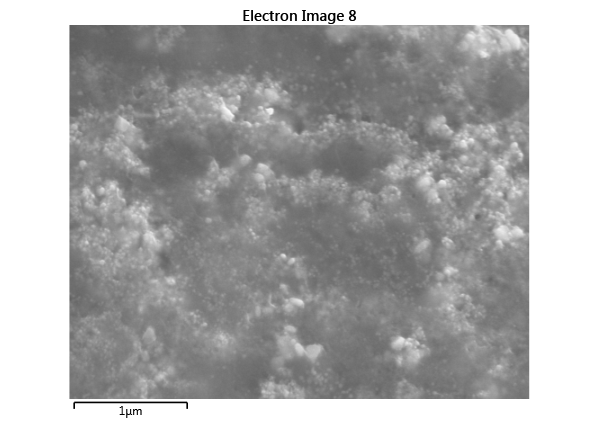


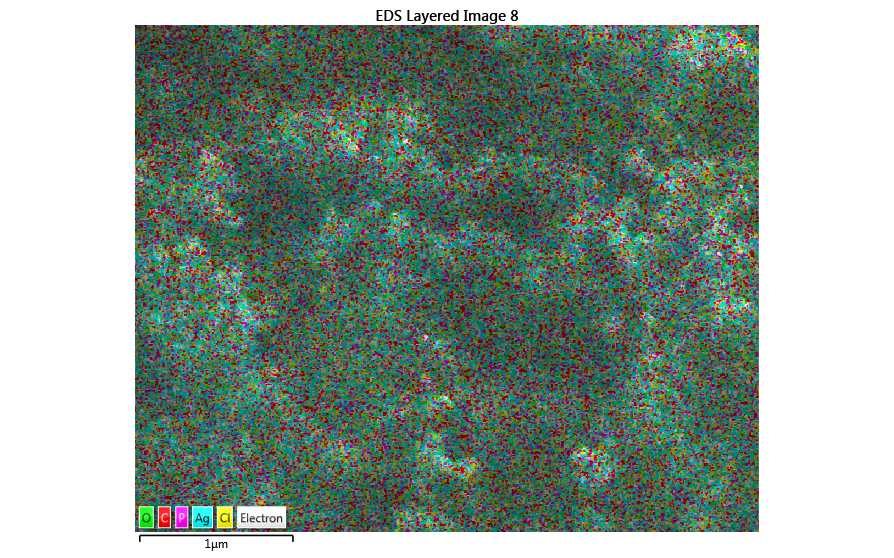


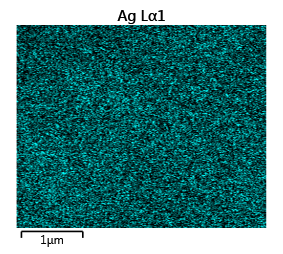

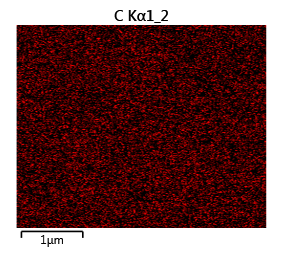

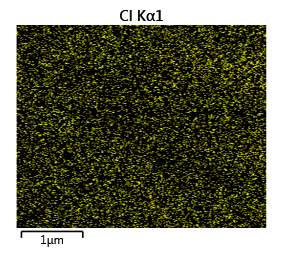

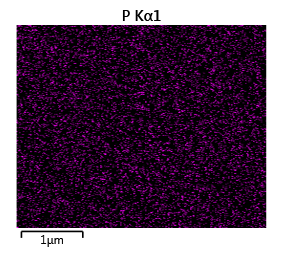

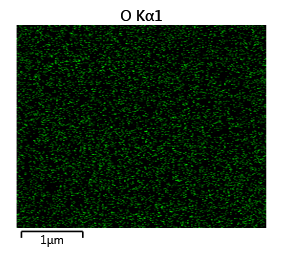

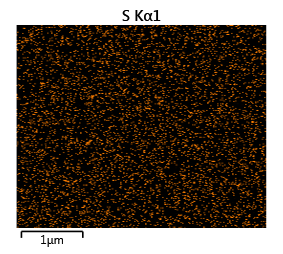

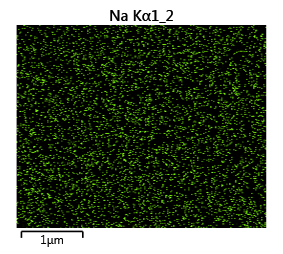

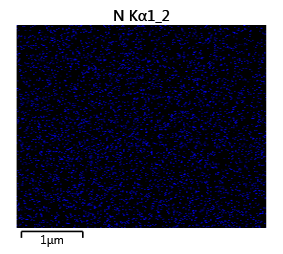


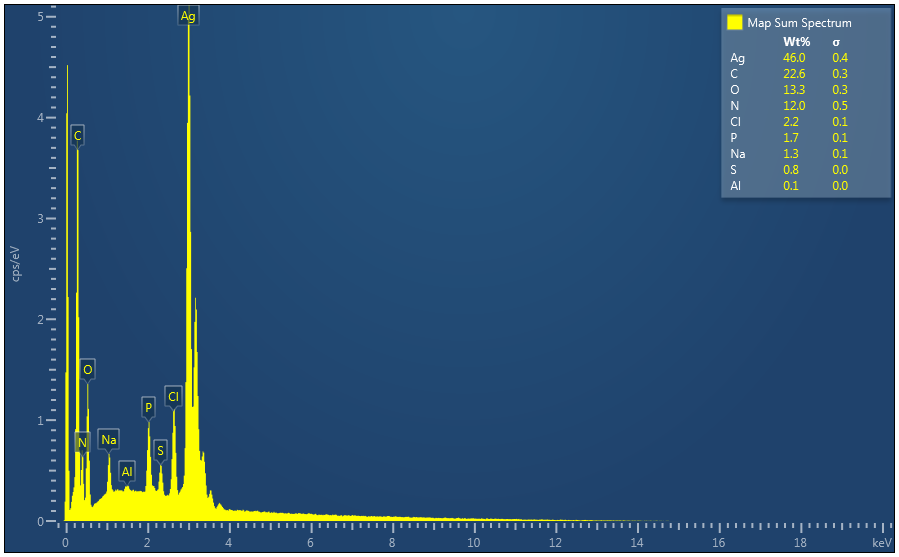


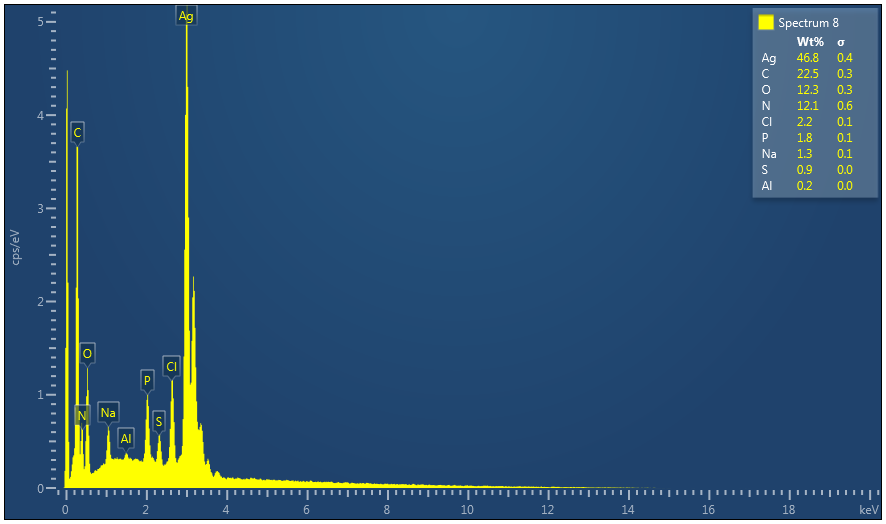


| Element | Line Type | Apparent Concentration | k Ratio | Wt% | Wt% Sigma | Standard Label | Factory Standard | Standard Calibration Date |
| --- | --- | --- | --- | --- | --- | --- | --- | --- |
| C | K series | 1.38 | 0.01385 | 22.48 | 0.32 | C Vit | Yes |  |
| N | K series | 1.26 | 0.00224 | 12.09 | 0.63 | BN | Yes |  |
| O | K series | 0.69 | 0.00233 | 12.28 | 0.32 | SiO2 | Yes |  |
| Na | K series | 0.15 | 0.00063 | 1.26 | 0.07 | Albite | Yes |  |
| Al | K series | 0.02 | 0.00013 | 0.15 | 0.04 | Al2O3 | Yes |  |
| P | K series | 0.34 | 0.00191 | 1.78 | 0.06 | GaP | Yes |  |
| S | K series | 0.12 | 0.00107 | 0.91 | 0.05 | FeS2 | Yes |  |
| Cl | K series | 0.30 | 0.00266 | 2.24 | 0.07 | NaCl | Yes |  |
| Ag | L series | 5.06 | 0.05061 | 46.80 | 0.45 | Ag | Yes |  |
| Total: |  |  |  | 100.00 |  |  |  |  |
