# Supplementary figures and images for: Sustainable biosynthesis of silver nanoparticles from vinegar bacteria fermentation waste: characterization, bioactivity and food packaging potential
Source: Sci Rep. 2026 May 14;16:22000. doi: 10.1038/s41598-026-53384-9 (PMC13365466; doi:10.1038/s41598-026-53384-9)

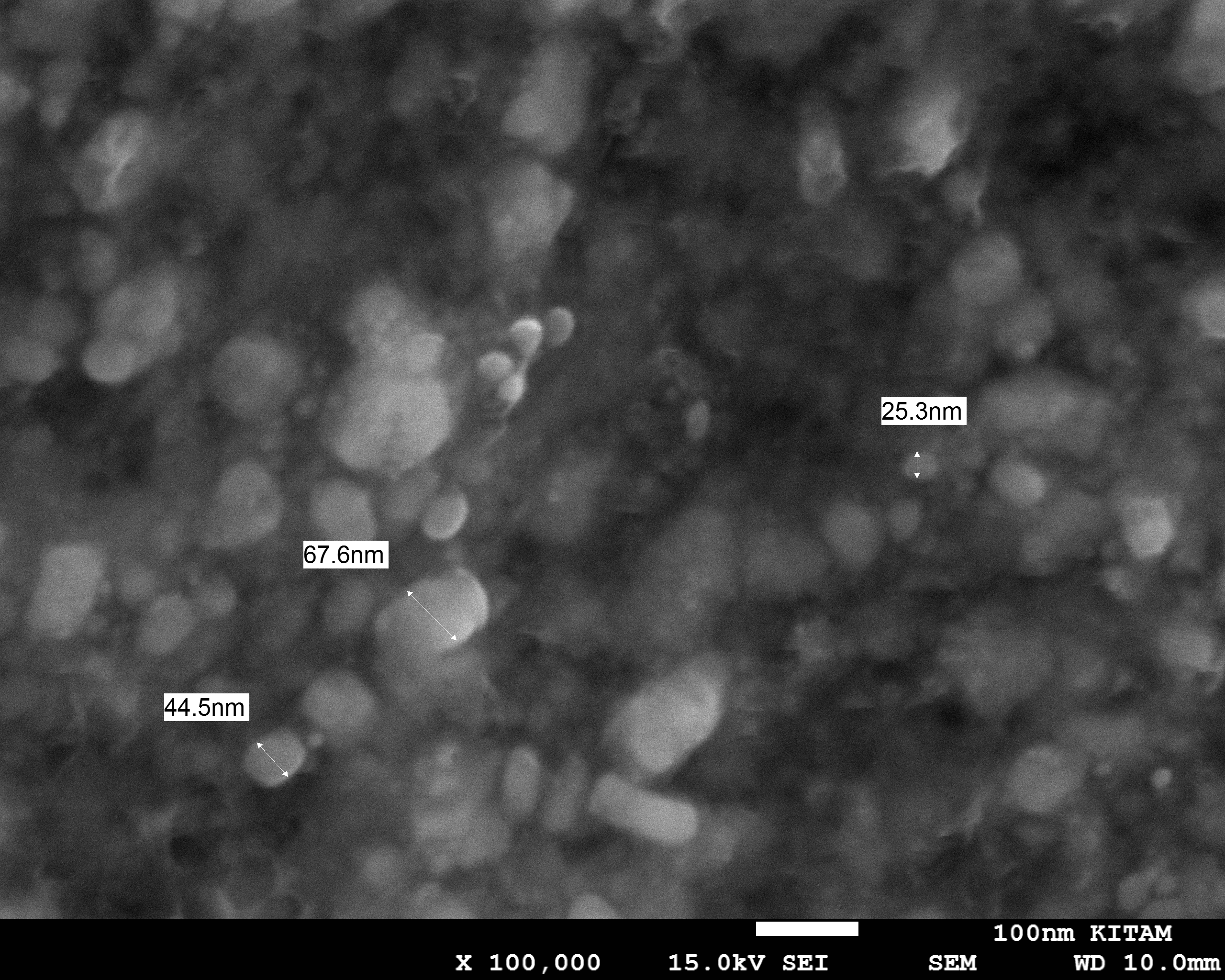

Supplement: Supplementary file 4 — Supplementary Material 4 [file 41598_2026_53384_MOESM4_ESM.zip › SEM Scala/1A_1.bmp]

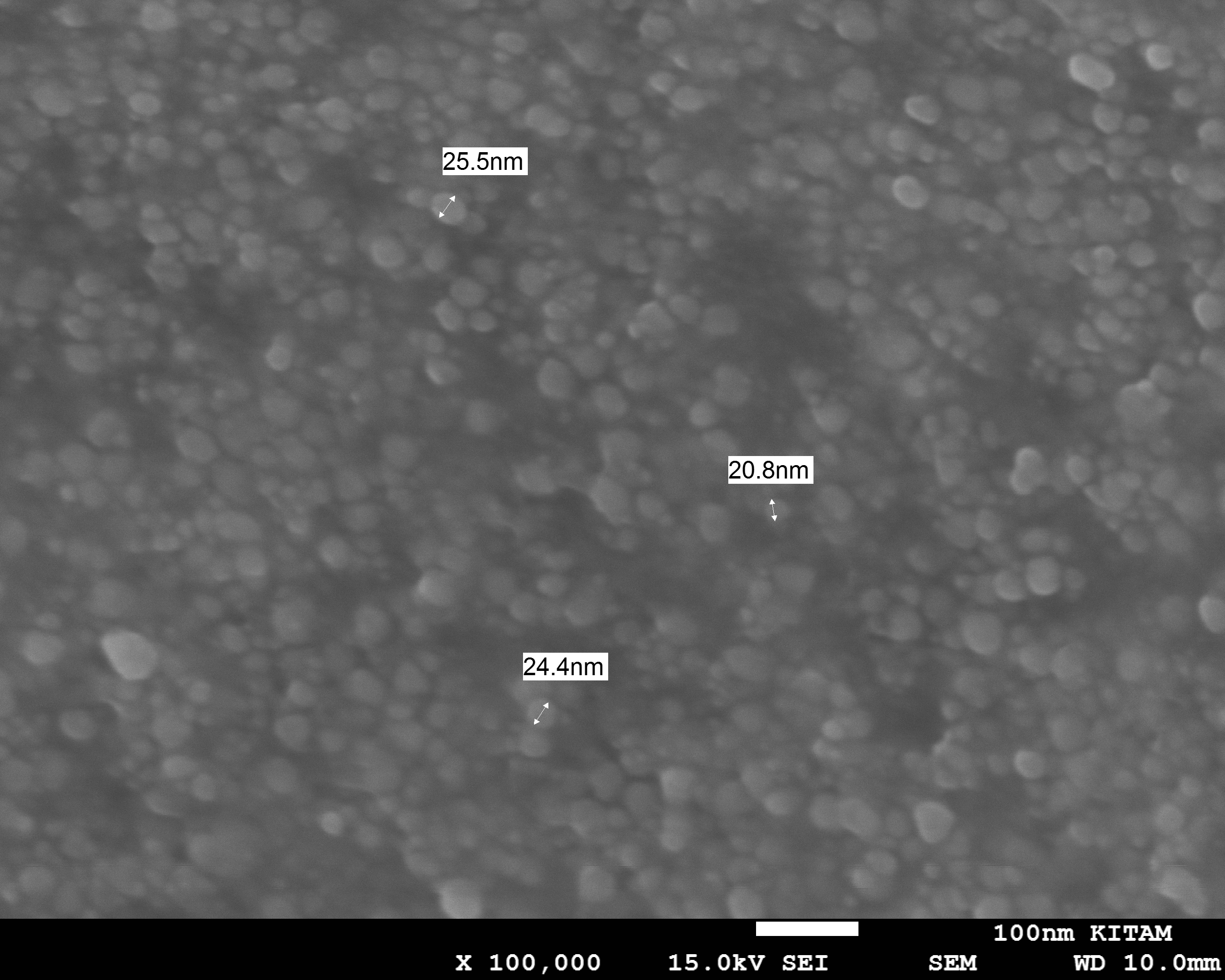

Supplement: Supplementary file 4 — Supplementary Material 4 [file 41598_2026_53384_MOESM4_ESM.zip › SEM Scala/1B_1.bmp]

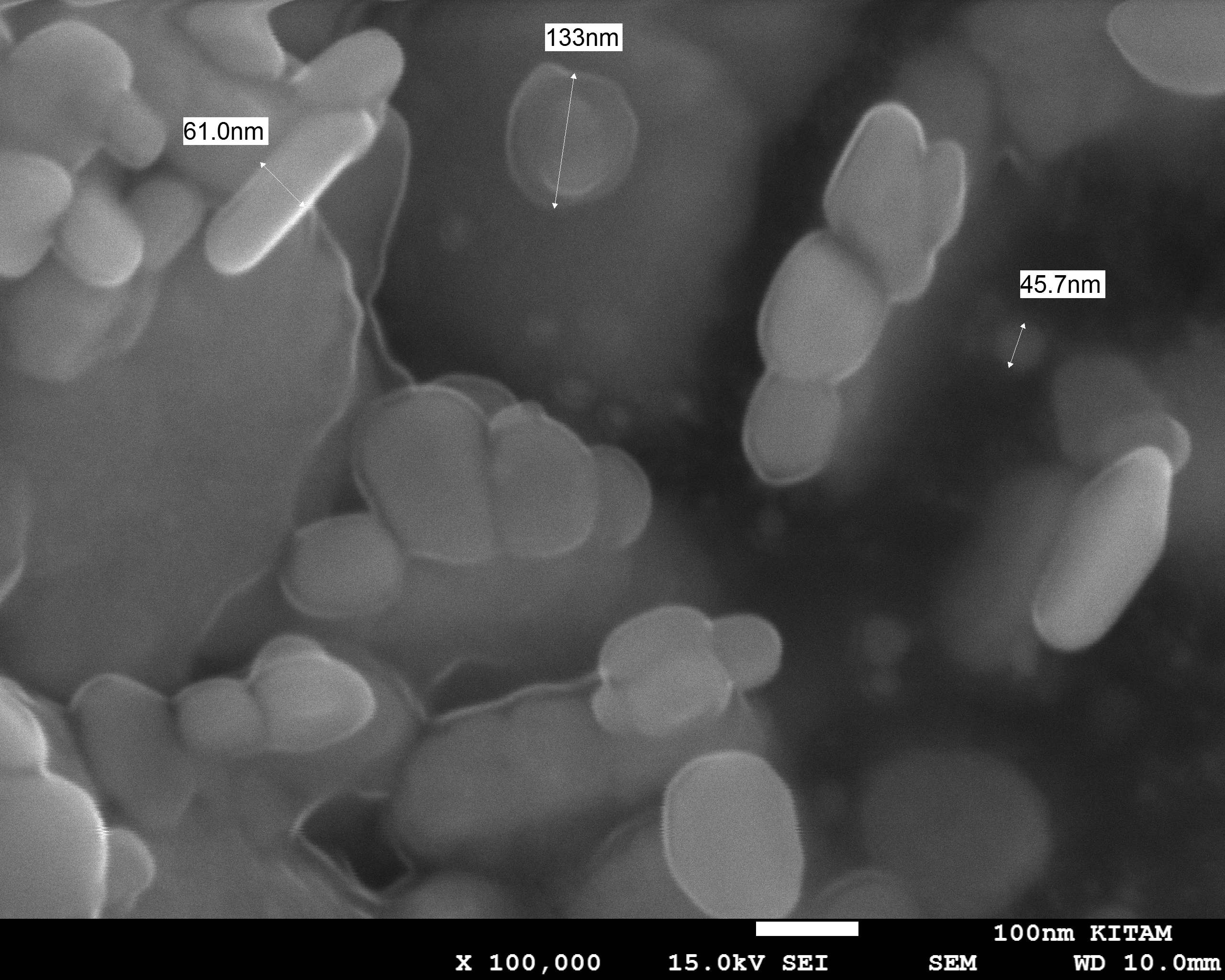

Supplement: Supplementary file 4 — Supplementary Material 4 [file 41598_2026_53384_MOESM4_ESM.zip › SEM Scala/2B_1.bmp]

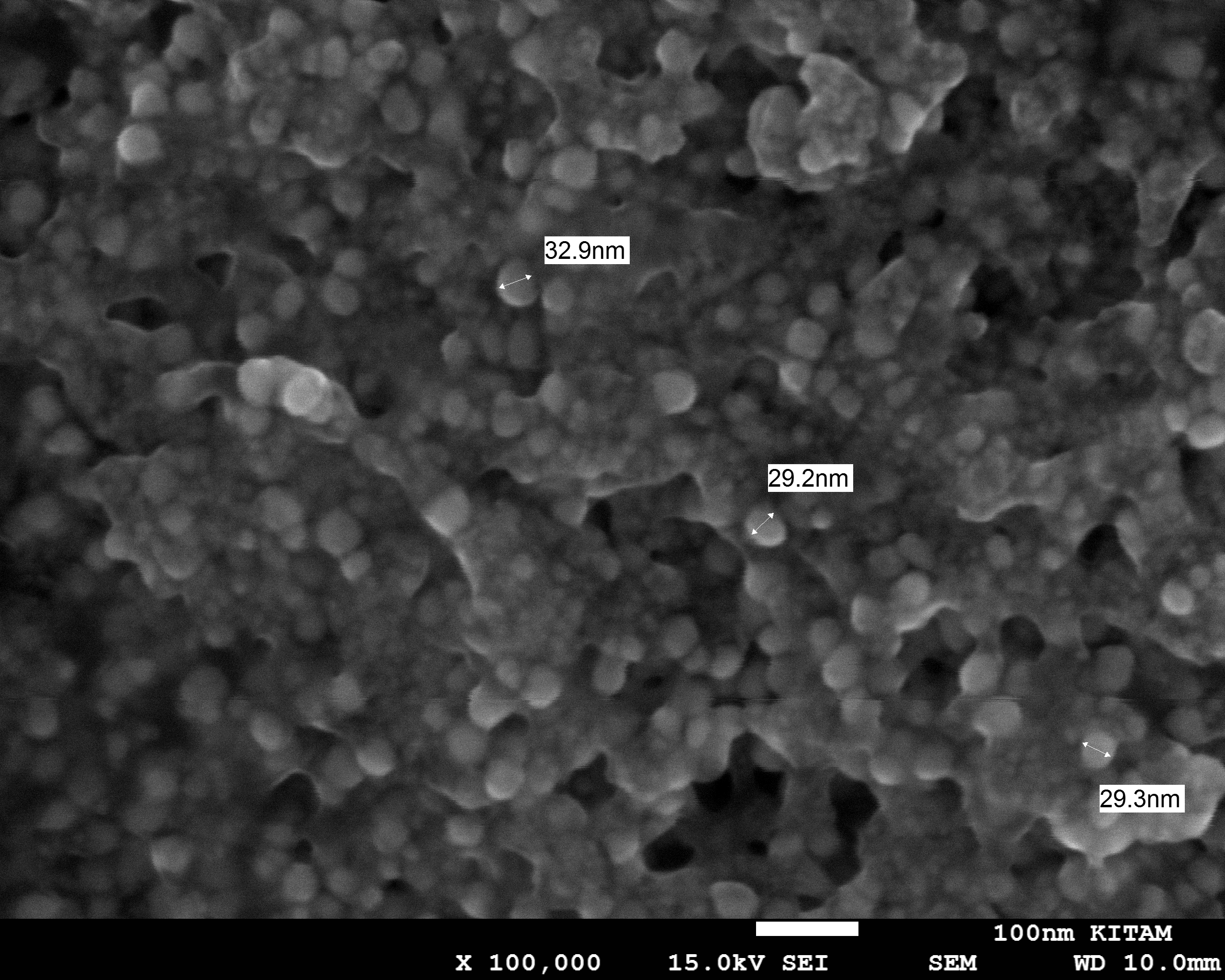

Supplement: Supplementary file 4 — Supplementary Material 4 [file 41598_2026_53384_MOESM4_ESM.zip › SEM Scala/2D_1.bmp]

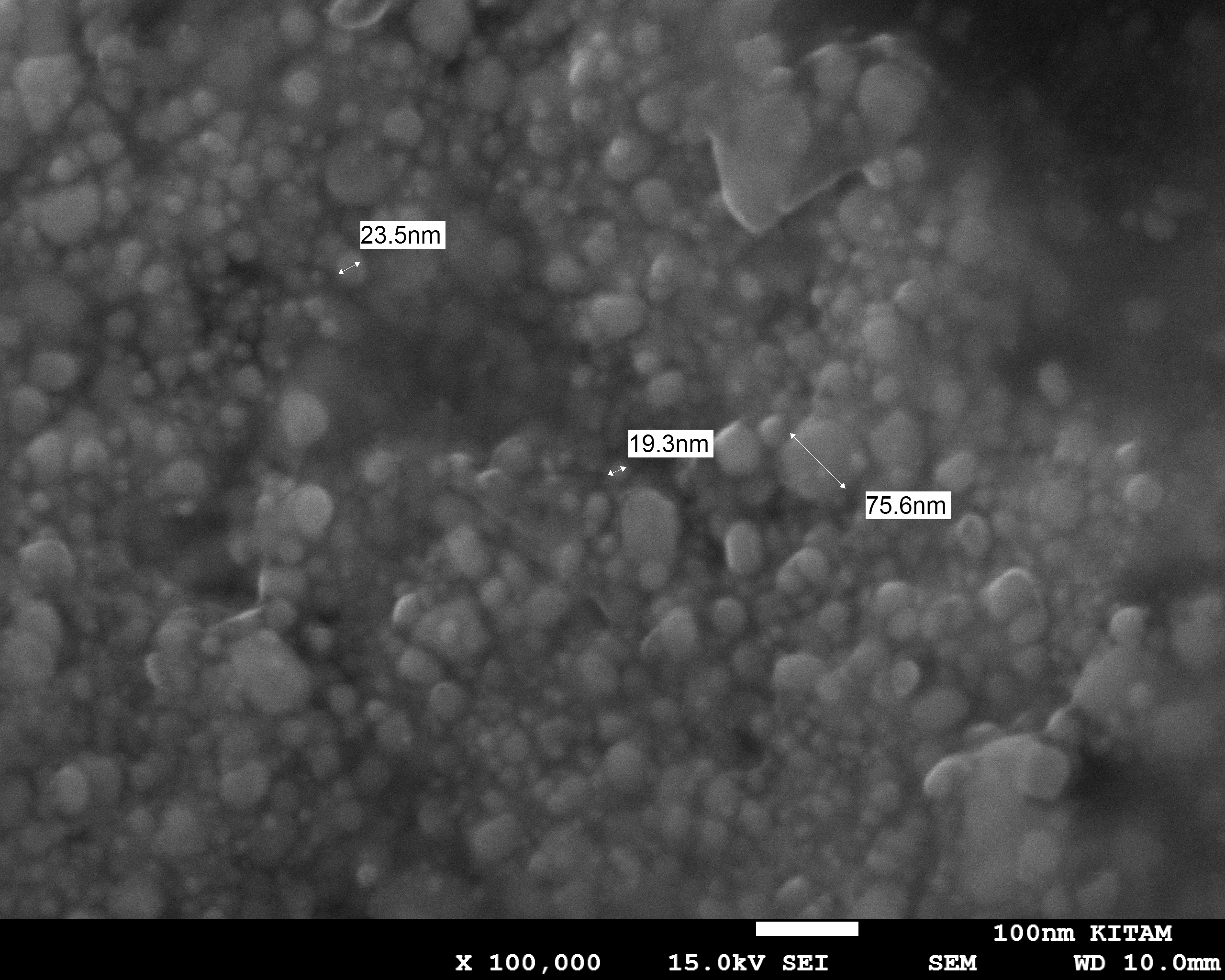

Supplement: Supplementary file 4 — Supplementary Material 4 [file 41598_2026_53384_MOESM4_ESM.zip › SEM Scala/3A_1.bmp]

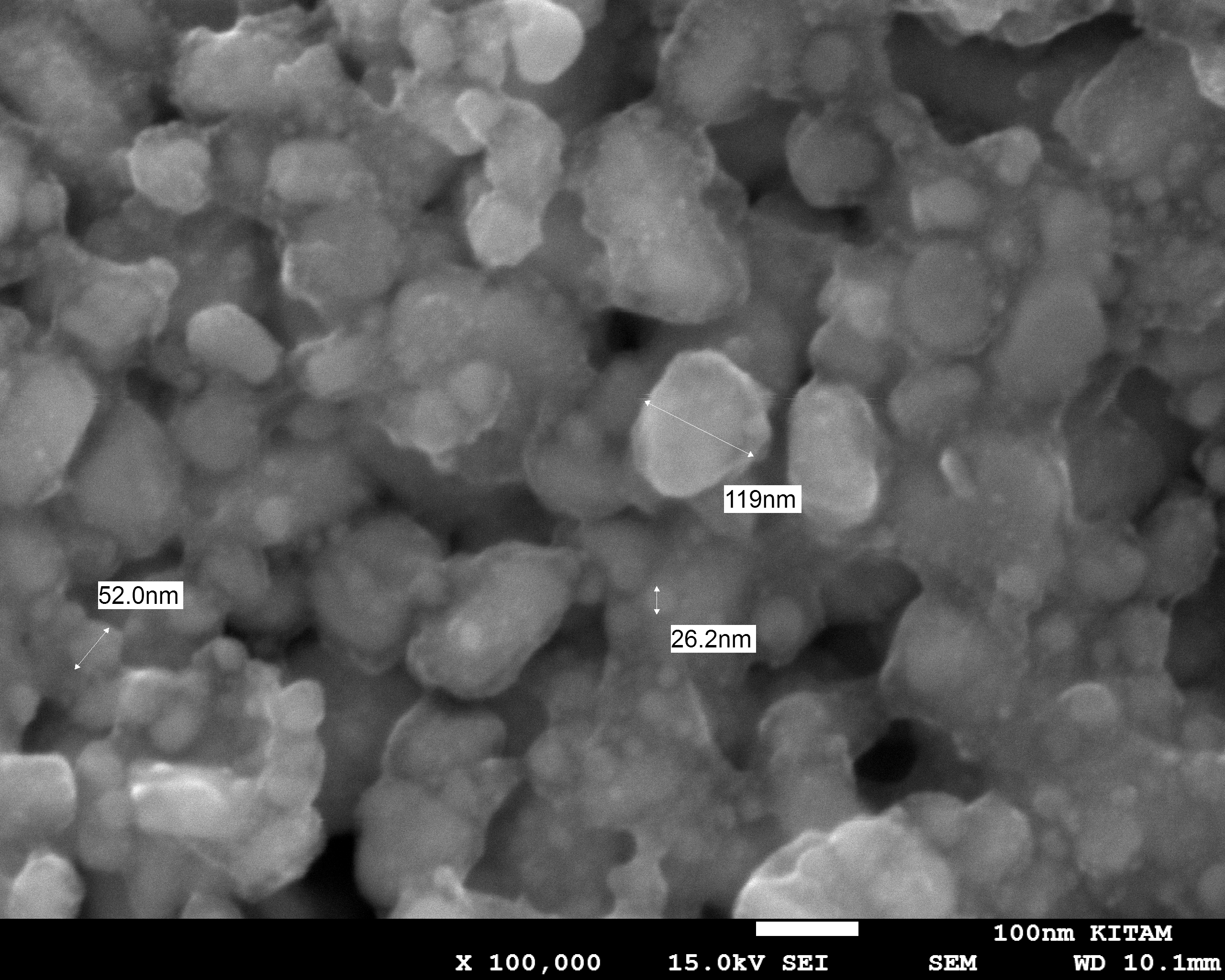

Supplement: Supplementary file 4 — Supplementary Material 4 [file 41598_2026_53384_MOESM4_ESM.zip › SEM Scala/X1_1.bmp]
