# Supplementary material for: Sustainable biosynthesis of silver nanoparticles from vinegar bacteria fermentation waste: characterization, bioactivity and food packaging potential
Source: Sci Rep. 2026 May 14;16:22000. doi: 10.1038/s41598-026-53384-9 (PMC13365466; doi:10.1038/s41598-026-53384-9)
Supplement: Supplementary file 5 — Supplementary Material 5 [file 41598_2026_53384_MOESM5_ESM.docx]

**SUPPLEMENTARY MATERIALS**

**Sustainable Biosynthesis of Silver Nanoparticles from Vinegar Bacteria Fermentation Waste: Characterization, Bioactivity and Food Packaging Potential**

Perihan Akbaş^a^*, Ali Osman Adıgüzel^b^, Elife Kaya^c^, Serpil Könen Adıgüzel^d^, Eda Uğurtay^e^

**Supplementary material 1.** Table showing similarities between the 16S rRNA gene sequences of isolate 1A and those of closely related species.

| Taxon Name | Accession number | Similarity | Variation ratio |
| --- | --- | --- | --- |
| *Acetobacter pasteurianus* subsp. pasteurianus LMG 1262 | BACG01000075 | 99.77 | 3/1331 |
| *Acetobacter oryzoeni* B6 | MK367404 | 99.77 | 3/1330 |
| *Acetobacter pasteurianus* subsp. paradoxus LMG 1591 | CP015168 | 99.70 | 4/1331 |
| *Acetobacter oryzifermentans* SLV-7 | CP011120 | 99.70 | 4/1331 |
| *Acetobacter ascendens* LMG 1590 | CP015164 | 99.62 | 5/1331 |
| Acetobacter *pomorum* LHT2458 | AJ001632 | 99.55 | 6/1331 |
| *Acetobacter vaccinii* C17-3 | MN121517 | 98.05 | 26/1331 |
| *Acetobacter syzygii* 9H-2 | BAMZ01000045 | 97.97 | 27/1331 |
| *Acetobacter okinawensis* JCM 25146 | BAJU01000075 | 97.97 | 27/1331 |
| *Acetobacter ghanensis* 430A | EF030713 | 97.90 | 28/1331 |
| *Acetobacter lambici* LMG 27439 | HF969863 | 97.90 | 28/1331 |
| Acetobacter *peroxydans* ATCC 12874 | jgi.1047262 | 97.66 | 31/1327 |
| *Acetobacter lovaniensis* LMG 1617 | AJ419837 | 97.60 | 32/1331 |
| *Acetobacter fabarum* 985 | AM905849 | 97.60 | 32/1331 |
| *Acetobacter papaya* JCM 25143 | BAIN01000105 | 97.36 | 35/1326 |

**Supplementary material 2.** Table showing the similarities between the 16S rRNA gene sequences of isolate 1B and those of closely related species.

| Taxon Name | Accession number | Similarity | Variation ratio |
| --- | --- | --- | --- |
| *Acetobacter pasteurianus* subsp. pasteurianus LMG 1262 | BACG01000075 | 99.92 | 1/1300 |
| *Acetobacter oryzoeni* B6 | MK367404 | 99.92 | 1/1299 |
| *Acetobacter pasteurianus* subsp. paradoxus LMG 1591 | CP015168 | 99.85 | 2/1300 |
| *Acetobacter oryzifermentans* SLV-7 | CP011120 | 99.85 | 2/1300 |
| *Acetobacter ascendens* LMG 1590 | CP015164 | 99.77 | 3/1300 |
| Acetobacter *pomorum* LHT2458 | AJ001632 | 99.69 | 4/1300 |
| *Acetobacter syzygii* 9H-2 | BAMZ01000045 | 98.23 | 23/1300 |
| *Acetobacter okinawensis* JCM 25146 | BAJU01000075 | 98.23 | 23/1300 |
| *Acetobacter ghanensis* 430A | EF030713 | 97.55 | 33/1348 |
| *Acetobacter lambici* LMG 27439 | HF969863 | 97.55 | 33/1348 |
| Acetobacter vaccinii C17-3 | MN121517 | 98.15 | 24/1300 |
| *Acetobacter lovaniensis* LMG 1617 | AJ419837 | 97.85 | 28/1300 |
| *Acetobacter suratthaniensis AI32* | AB937774 | 97.42 | 34/1317 |
| *Acetobacter fabarum* 985 | AM905849 | 97.85 | 28/1300 |
| Acetobacter peroxydans ATCC 12874 | jgi.1047262 | 97.76 | 29/1296 |

**Supplementary material 3.** Table showing the similarities between the 16S rRNA gene sequences of isolate 2B and those of closely related species.

| Taxon Name | Accession number | Similarity | Variation ratio |
| --- | --- | --- | --- |
| *Acetobacter pasteurianus* subsp. pasteurianus LMG 1262 | BACG01000075 | 99.85 | 2/1348 |
| *Acetobacter oryzoeni* B6 | MK367404 | 99.85 | 2/1347 |
| *Acetobacter pasteurianus* subsp. paradoxus LMG 1591 | CP015168 | 99.78 | 3/1348 |
| *Acetobacter oryzifermentans* SLV-7 | CP011120 | 99.78 | 3/1348 |
| *Acetobacter ascendens* LMG 1590 | CP015164 | 99.70 | 4/1348 |
| Acetobacter pomorum LHT2458 | AJ001632 | 99.63 | 5/1348 |
| *Acetobacter vaccinii* C17-3 | MN121517 | 98.15 | 25/1348 |
| Acetobacter peroxydans ATCC 12874 | jgi.1047262 | 97.77 | 30/1344 |
| *Acetobacter syzygii* 9H-2 | BAMZ01000045 | 97.63 | 32/1348 |
| *Acetobacter okinawensis* JCM 25146 | BAJU01000075 | 97.63 | 32/1348 |
| *Acetobacter ghanensis* 430A | EF030713 | 97.55 | 33/1348 |
| *Acetobacter lambici* LMG 27439 | HF969863 | 97.55 | 33/1348 |
| *Acetobacter suratthaniensis AI32* | AB937774 | 97.42 | 34/1317 |
| *Acetobacter papaya* JCM 25143 | BAIN01000105 | 97.32 | 36/1343 |
| *Acetobacter lovaniensis* LMG 1617 | AJ419837 | 97.26 | 37/1348 |

**Supplementary material 4.** Table showing the similarities between the 16S rRNA gene sequences of isolate 2D and those of closely related species.

| Taxon Name | Accession number | Similarity | Variation ratio |
| --- | --- | --- | --- |
| *Acetobacter pasteurianus* subsp. pasteurianus LMG 1262 | BACG01000075 | 99.85 | 2/1330 |
| *Acetobacter oryzoeni* B6 | MK367404 | 99.85 | 2/1329 |
| *Acetobacter pasteurianus* subsp. paradoxus LMG 1591 | CP015168 | 99.77 | 3/1330 |
| *Acetobacter oryzifermentans* SLV-7 | CP011120 | 99.77 | 3/1330 |
| *Acetobacter ascendens* LMG 1590 | CP015164 | 99.70 | 4/1330 |
| Acetobacter pomorum LHT2458 | AJ001632 | 99.62 | 5/1330 |
| *Acetobacter vaccinii* C17-3 | MN121517 | 98.12 | 25/1330 |
| *Acetobacter syzygii* 9H-2 | BAMZ01000045 | 97.97 | 27/1330 |
| *Acetobacter okinawensis* JCM 25146 | BAJU01000075 | 97.97 | 27/1330 |
| *Acetobacter ghanensis* 430A | EF030713 | 97.89 | 28/1330 |
| *Acetobacter lambici* LMG 27439 | HF969863 | 97.89 | 28/1330 |
| Acetobacter peroxydans ATCC 12874 | jgi.1047262 | 97.74 | 30/1326 |
| *Acetobacter lovaniensis LMG 1617* | AJ419837 | 97.59 | 32/1330 |
| *Acetobacter fabarum 985* | AM905849 | 97.59 | 32/1330 |
| *Acetobacter suratthaniensis* AI32 | AB937774 | 97.43 | 34/1324 |

**Supplementary material 5.** Table showing the similarities between the 16S rRNA gene sequences of isolate 3A and those of closely related species.

| Taxon Name | Accession number | Similarity | Variation ratio |
| --- | --- | --- | --- |
| *Acetobacter pasteurianus* subsp. pasteurianus LMG 1262 | BACG01000075 | 99.48 | 7/1354 |
| *Acetobacter oryzoeni* B6 | MK367404 | 99.48 | 7/1353 |
| *Acetobacter pasteurianus* subsp. paradoxus LMG 1591 | CP015168 | 99.41 | 8/1354 |
| *Acetobacter oryzifermentans* SLV-7 | CP011120 | 99.41 | 8/1354 |
| *Acetobacter ascendens* LMG 1590 | CP015164 | 99.34 | 9/1354 |
| Acetobacter pomorum LHT2458 | AJ001632 | 99.26 | 10/1354 |
| Acetobacter vaccinii C17-3 | MN121517 | 97.78 | 30/1354 |
| *Acetobacter peroxydans ATCC 12874* | jgi.1047262 | 97.41 | 35/1350 |
| Acetobacter syzygii 9H-2 | BAMZ01000045 | 97.34 | 36/1355 |
| Acetobacter okinawensis JCM 25146 | BAJU01000075 | 97.34 | 36/1355 |
| Acetobacter ghanensis 430A | EF030713 | 97.27 | 37/1355 |
| Acetobacter lambici LMG 27439 | HF969863 | 97.27 | 37/1355 |
| *Acetobacter suratthaniensis AI32* | AB937774 | 97.13 | 38/1323 |
| *Acetobacter lovaniensis LMG 1617* | AJ419837 | 96.97 | 41/1355 |
| Acetobacter fabarum 985 | AM905849 | 96.97 | 41/1355 |

**Supplementary material 6.** Table showing the similarities between the 16S rRNA gene sequences of isolate X1 and those of closely related species.

| Taxon Name | Accession number | Similarity | Variation ratio |
| --- | --- | --- | --- |
| *Komagataeibacter* *melaceti* AV382 | MT422125 | 99.10 | 12/1333 |
| *Komagataeibacter* *nataicola* LMG 1536 | AB166743 | 98.72 | 17/1333 |
| *Komagataeibacter* *sucrofermentans* LMG 18788 | AJ007698 | 98.72 | 17/1333 |
| *Komagataeibacter* *kakiaceti* JCM 25156 | BAIO01000137 | 98.72 | 17/1333 |
| *Komagataeibacter* *saccharivorans* LMG 1582 | AB166740 | 98.72 | 17/1333 |
| *Komagataeibacter* *rhaeticus* DST GL02 | AY180961 | 98.67 | 17/1276 |
| *Komagataeibacter* *europaeus* DSM 6160 | Z21936 | 98.65 | 18/1333 |
| *Komagataeibacter swingsii* DST GL01 | AY180960 | 98.65 | 18/1333 |
| *Komagataeibacter* *xylinus* NBRC 15237 | BCTP01000094 | 98.57 | 19/1333 |
| *Komagataeibacter* *diospyri* MSKU9 | BDLU01000102 | 98.57 | 19/1333 |
| *Komagataeibacter* *melomenusus* AV436 | MT422127 | 98.50 | 20/1333 |
| *Komagataeibacter* *intermedius* TF2 | BANF01000022 | 98.42 | 21/1333 |
| *Komagataeibacter oboediens* DSM 11826 | AB205221 | 98.42 | 21/1333 |
| *Komagataeibacter medellinensis NBRC 3288* | AP012159 | 98.05 | 26/1333 |
| *Novacetimonas* *pomaceti* T5K1 | MH355952 | 97.96 | 26/1276 |
